# Effect of Alpha Linolenic Acid Supplementation on Serum Prostate Specific Antigen (PSA): Results from the Alpha Omega Trial

**DOI:** 10.1371/journal.pone.0081519

**Published:** 2013-12-11

**Authors:** Ingeborg A. Brouwer, Johanna M. Geleijnse, Veronique M. Klaasen, Liesbeth A. Smit, Erik J. Giltay, Janette de Goede, Annemieke C. Heijboer, Daan Kromhout, Martijn B. Katan

**Affiliations:** 1 Department of Health Sciences and the EMGO+ Institute for Health and Care Research, Faculty of Earth and Life Sciences, VU University Amsterdam, Amsterdam, The Netherlands; 2 Division of Human Nutrition, Wageningen University, Wageningen, The Netherlands; 3 Department of Psychiatry, Leiden University Medical Center, Leiden, The Netherlands; 4 Endocrine Laboratory, Department of Clinical Chemistry, VU University Medical Center, Amsterdam, The Netherlands; Cardiff University, United Kingdom

## Abstract

**Background:**

Alpha linolenic acid (ALA) is the major omega-3 fatty acid in the diet. Evidence on health effects of ALA is not conclusive, but some observational studies found an increased risk of prostate cancer with higher intake of ALA. We examined the effect of ALA supplementation on serum concentrations of prostate-specific antigen (PSA), a biomarker for prostate cancer.

**Methods:**

The Alpha Omega Trial (ClinicalTrials.gov Identifier: NCT00127452) was a double-blind, placebo-controlled trial of ALA and the fish fatty acids eicosapentanoic acid (EPA) and docosahexanoic acid (DHA) on the recurrence of cardiovascular disease, using a 2×2 factorial design. Blood was collected at the start and the end of the intervention period. The present analysis included 1622 patients with a history of a myocardial infarction, aged 60–80 years with an initial PSA concentration <4 ng/mL. They received either 2 g per day of ALA or placebo in margarine spreads for 40 months. T-tests and logistic regression were used to assess the effects of ALA supplementation on changes in serum PSA (both continuously and as a dichotomous outcome, cut-off point: >4 ng/mL).

**Findings:**

Mean serum PSA increased by 0.42 ng/mL on placebo (n = 815) and by 0.52 ng/mL on ALA (n = 807), a difference of 0.10 (95% confidence interval: −0.02 to 0.22) ng/mL (P = 0·12). The odds ratio for PSA rising above 4 ng/mL on ALA versus placebo was 1.15 (95% CI: 0.84–1.58).

**Interpretation:**

An additional amount of 2 g of ALA per day increased PSA by 0.10 ng/mL, but the confidence interval ranged from −0.02 to 0.22 ng/mL and included no effect. Therefore, more studies are needed to establish whether or not ALA intake has a clinically significant effect on PSA or prostate cancer.

**Trial registration information:**

ClinicalTrials.gov; Identifier: NCT00127452. URL: http://www.clinicaltrials.gov/ct2/show/NCT00127452.

## Introduction

Alpha linolenic acid (ALA, C18∶3 n-3) is an essential omega-3 fatty acid and it is the precursor of the other long chain omega-3 fatty acids. Human tissues can convert ALA into the longer chain omega-3 fatty acids eicosapentaenoic acid (EPA, C20∶5 n-3) and docosahexaenoic acid (DHA C22∶6 n-3), but only to a limited extent. [1], [2] Major dietary sources of alpha-linolenic acid are soybean oil, canola oil and walnuts. Flaxseed is particularly rich in alpha-linolenic acid and capsules of flaxseed oil are sold as supplements. The average dietary intake of ALA in high-income countries is about 2 g per day. [3].

Observational studies suggest that an increased intake of alpha-linolenic acid is associated with a moderately lower risk of cardiovascular disease. [4] In contrast, a higher intake of alpha-linolenic acid has also been suggested to be associated with a higher risk of prostate cancer. Two meta-analyses that included both prospective and case control studies found that higher intakes of alpha-linolenic acid and higher levels in blood and adipose tissue were associated with increased risk of prostate cancer. [5], [6], [7] Simon et al. observed that higher concentrations of ALA in blood or adipose tissue were associated with a higher risk of prostate cancer (relative risk [RR] = 1.54; 95% CI 1.16–2.06), but no association was found for dietary ALA intake as assessed by food frequency questionnaire (RR = 1.09; 95% CI 0.91–1.32). [5] Carayol et al. limited their meta-analysis to prospective studies on dietary ALA and incident prostate cancer, and found no association (RR = 0.97; 95% CI 0.86–1.10). However, this meta-analysis showed a small significant adverse association when comparing high with low ALA intake categories [8] and another large observational study that was not yet included in the meta-analysis suggests a similar association. [9] We conclude from these results that the association of ALA with prostate cancer is not clear.

The Alpha Omega Trial was a double-blind placebo-controlled trial in 60–80 year-old patients with a history of myocardial infarction who received moderate additional amounts of omega-3 fatty acids for the prevention of recurrent cardiovascular diseases. The incidence of prostate cancer was monitored as a potential adverse effect in this study. [10], [11] ALA supplementation was not related to the incidence of prostate cancer. However, there were only 42 incident prostate cancer cases and thus the power to detect an effect was low. [10], [11] Serum concentrations of prostate specific antigen (PSA), a serine protease produced by prostatic epithelial cells [12], are often elevated in men with prostate cancer (www.cancer.gov/cancertopics/factsheets/detection/PSA). High concentrations may predict long-term increases in prostate cancer incidence and mortality. [13] Here we report the effect of an additional amount of ALA of 2 grams daily on serum PSA concentrations in older patients who had suffered a myocardial infarction and had participated in the Alpha Omega Trial. [11].

## Methods

### Study Population

**Figure 1:**
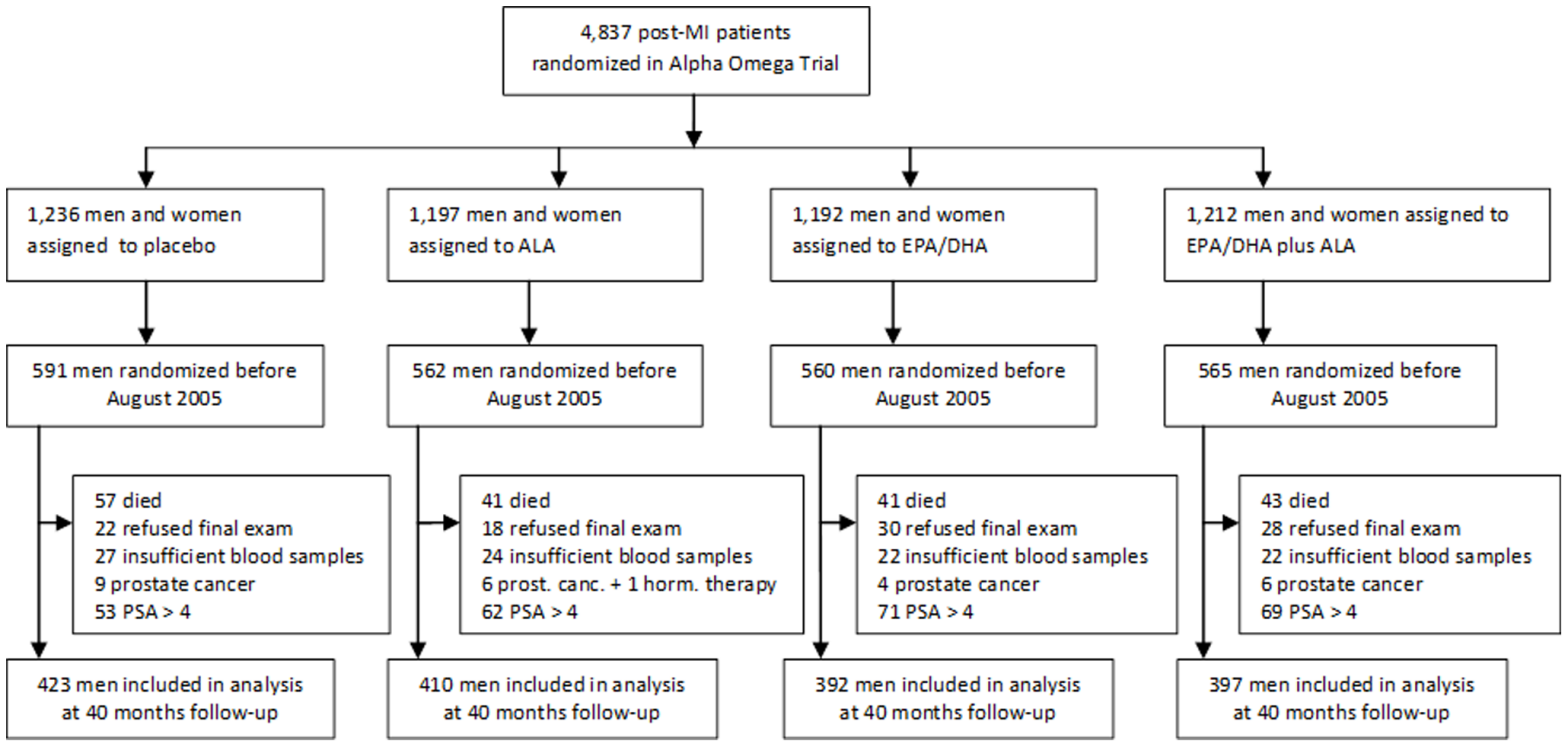
Participant flow for the study on effect of alpha linolenic acid (ALA) supplementation on PSA in post myocardial patients in the Alpha Omega Trial.

The Alpha Omega Trial has been described in detail previously. [10], [11] The protocol for this trial and supporting CONSORT checklist are available as supporting information; see Checklist S1 and Protocol S1. The participants were 3783 men and 1054 women who had suffered a myocardial infarction 10 years or less prior to randomization. The present study was limited to 2278 men randomized before August 2005 (Figure 1). We did not collect final blood samples for men randomized after that date because of a lack of funds. We excluded data of men who had prostate cancer at baseline or who used androgenic or anti-androgenic medication, who died during the intervention period, who refused final examinations, or for whom not enough blood was available. We only included patients with a baseline PSA concentration <4 ng/mL, because patients with a PSA level above 4 ng/mL have a high chance of having undiagnosed prostate cancer (21.3%) or of having hyperplasia or prostatitis; this may influence the effect of alpha linolenic acid on PSA. [13] This left a study population of 1622 men (Figure 1). The patients provided written informed consent. The trial was approved by a central medical ethics committee (Haga Hospital Leyenburg, The Hague, The Netherlands) and by local ethics committees of participating hospitals.

### Intervention with Omega-3 Fatty Acids

The patients were randomly assigned to receive trial margarines according to a 2-by-2 factorial design, for 40 months. For logistical reasons, all patients received a placebo margarine during the first 4–6 weeks after randomization. After this period the patients received either approximately 2 g ALA per day, or 400 mg EPA and DHA, or both, or a placebo with no omega-3 fatty acids. [10] The additional amount of ALA corresponded with the recommended dietary allowance. [3] In the trial margarines for the active treatment groups, the various omega-3 fatty acids replaced an equivalent amount of oleic acid in the margarine. Subjects were asked to consume approximately 20 grams of margarine per day. Margarines were identical in taste, texture, color, and odor. An objective measure of adherence was obtained by determining the proportions of fatty acids in plasma cholesteryl esters in a random subgroup of 217 male patients at baseline, 211 at 20 months and 523 at 40 months. [10].

### Data Collection and Follow-up Procedures

The patients underwent physical examinations by trained research nurses at baseline and after 40 months. Patients filled out questionnaires about demographic factors, lifestyle characteristics, medications and medical history. The definition of diabetes was based on a physician diagnosis, and/or the use of antidiabetic drugs and/or elevated plasma glucose levels. Obesity was defined as Body Mass Index (kg/m^2^)>30.

Blood samples were obtained at the subjects’ home or at a hospital. Tubes were sent via standard postal service to a central laboratory. In a pilot study on 76 patients, [10] 89% of blood samples was delivered within 24 h and 96% within 48 h. Serum was stored at −80°C for a maximum of 8 year. After the study had been completed we measured total PSA concentrations with an immunometric assay (Architect, Abbott Diagnostics, Abbott Park, Illinois USA) at the endocrine laboratory of VU University Amsterdam medical center. Laboratory personnel was blinded to the treatment groups. The detection limit was 0·1 ng/mL, the intra-assay coefficient of variation at a PSA level of 0.5, 4 and 25 ng/mL was 5%, 3% and 4%, respectively, and the inter-assay coefficient of variation 8%, 6%, and 6%, respectively.

### Statistical Analysis

Data were analyzed according to a predefined statistical analysis plan. The two groups that received ALA were combined (n = 807) and compared with the two groups that did not receive ALA (n = 815). The primary endpoints were the change in serum PSA during the 40-month intervention period (continuously) and the progression from PSA ≤4 ng/mL to PSA >4 ng/mL.

Differences in changes of PSA between treatments were assessed with two-sided t-tests for independent samples. We also stratified that analysis for treatment with EPA/DHA (yes/no). Our study did not have the objective to investigate the effect of EPA plus DHA, but we show the outcomes for completeness. The dichotomous outcome of progression to PSA level >4 ng/mL (yes/no) was analyzed by logistic regression. PSA velocity is frequently used for monitoring the risk of prostate cancer. [14], [15] In a post-hoc analysis, we calculated the additional outcome of PSA velocity defined as change in PSA per year, which was categorized using different cut-off points (>0.50, >0.75 and >1.00 ng/mL). The odds ratio for different PSA velocities between the combined ALA group and the combined placebo group were also calculated by logistic regression. SPSS software version 17.0 was used for all analyses.

## Results

### Descriptive Data

Baseline characteristics were similar among treatment groups for the 1622 men with an initial PSA level at or below 4 ng/mL (Table 1). The mean age of the patients was 68.0 y, 20.7% was obese and 17.5% had diabetes, 16.6% smoked and 78% used at least one alcoholic drink per week. Their median PSA-level was 1.09 (25^th^ to 75^th^ percentile = 0.61 to 1.97) ng/mL at entry.

**Table 1:** Baseline characteristics of 1622 male patients of the Alpha Omega Trial who were included in the study on PSA change, by treatment group*.

	Placebo	EPA-DHA	ALA	ALA+EPA-DHA
	N = 423	N = 392	N = 410	N = 397
**Age (yr)**	67·8±5·1	68·2±5·2	68·1±5·4	68·0±5·1
**Body Mass Index (kg/m^2^)**	27·7±3·7	27·6±3·2	27·5±3·1	27·5±3·2
**Obese** * **(%)**	22·0	21·9	19·3	19·4
**Diabetic** † **(%)**	19·4	16·6	16·6	17·4
**Intermediate or higher education** ‡ **(%)**	47·6	41·7	44·3	46·5
**Smoking status**				
** Never (%)**	9·9	16·1	12·7	11·1
** Former (%)**	73·3	67·3	69·5	73·5
** Current (%)**	16·8	16·6	17·8	15·4
**Alcohol use**				
** ≥1 glass/wk (%)**	79·0	78·3	76·3	78·5
** <1 glass/wk or past drinker (%)**	16·5	16·8	18·1	14·4
** Never (%)**	4·5	4·8	5·6	7·1
**Serum Prostate Specific Antigen (ng/mL)**	1·37±0·96	1·37±1·01	1·42±0·97	1·43±0·97

Data are reported as mean ± standard deviation (SD) or percentages (%).ALA = alpha linolenic acid, EPA = eicosapentanoic acid = EPA, DHA = docosahexanoic acid.

* Subjects with initial PSA concentrations >4 ng/mL (n = 255) were excluded, see Methods and Figure 1.

** Body mass index ≥30 kg/m^2^.

†Self-reported diabetes diagnosed by a physician and/or treatment with antidiabetic medication and/or elevated plasma glucose level.

‡High school at intermediate or higher level, or higher vocational education college or university.

**Figure 2:**
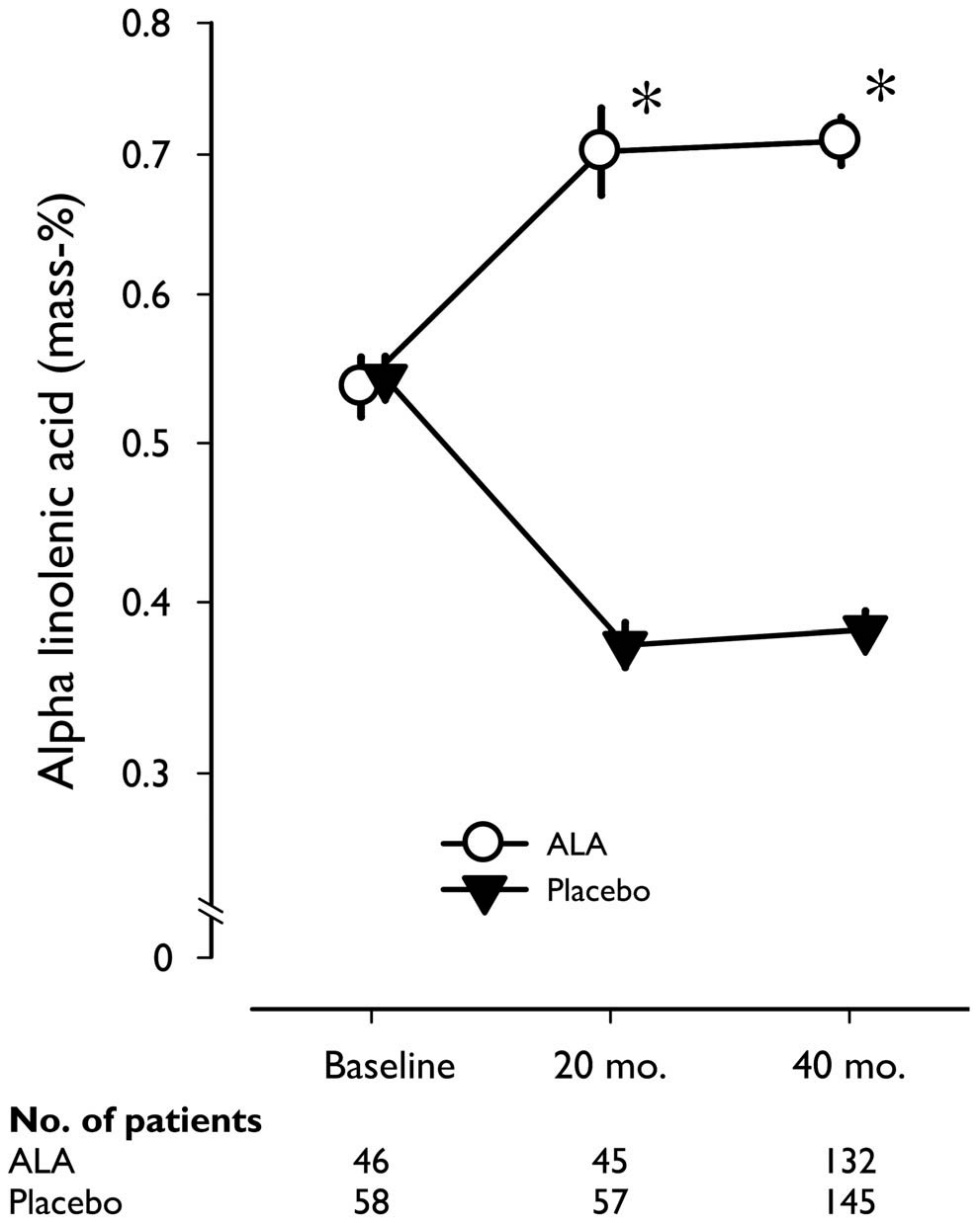
Alpha linolenic acid (ALA) concentrations in plasma cholesteryl esters at baseline, after 20 months and after 40 months, in random samples of post-MI patients, according to treatment group. Geometric mean values (expressed as mass percentage) on a logarithmic scale are given, with error bars indicating standard errors. After 20 and 40 months, ALA supplementation in the margarine increased serum ALA by 64·7% and 66·9% as compared with placebo. *P<0·001 for group difference at that time point, obtained by *t*-test for independent samples.

The average intake of trial margarine was 20.2 (SD: 3.5) grams per day and 97% of the participants consumed the margarine at least 80% of the time. The patients received on average an additional amount of 2 g of ALA per day. A high adherence was confirmed by the proportion of ALA in plasma cholesteryl esters, which increased by 65% after 20 months and 67% after 40 months in the ALA relative to the placebo group (Figure 2).

### Effect of ALA Supplementation on Serum PSA

**Figure 3:**
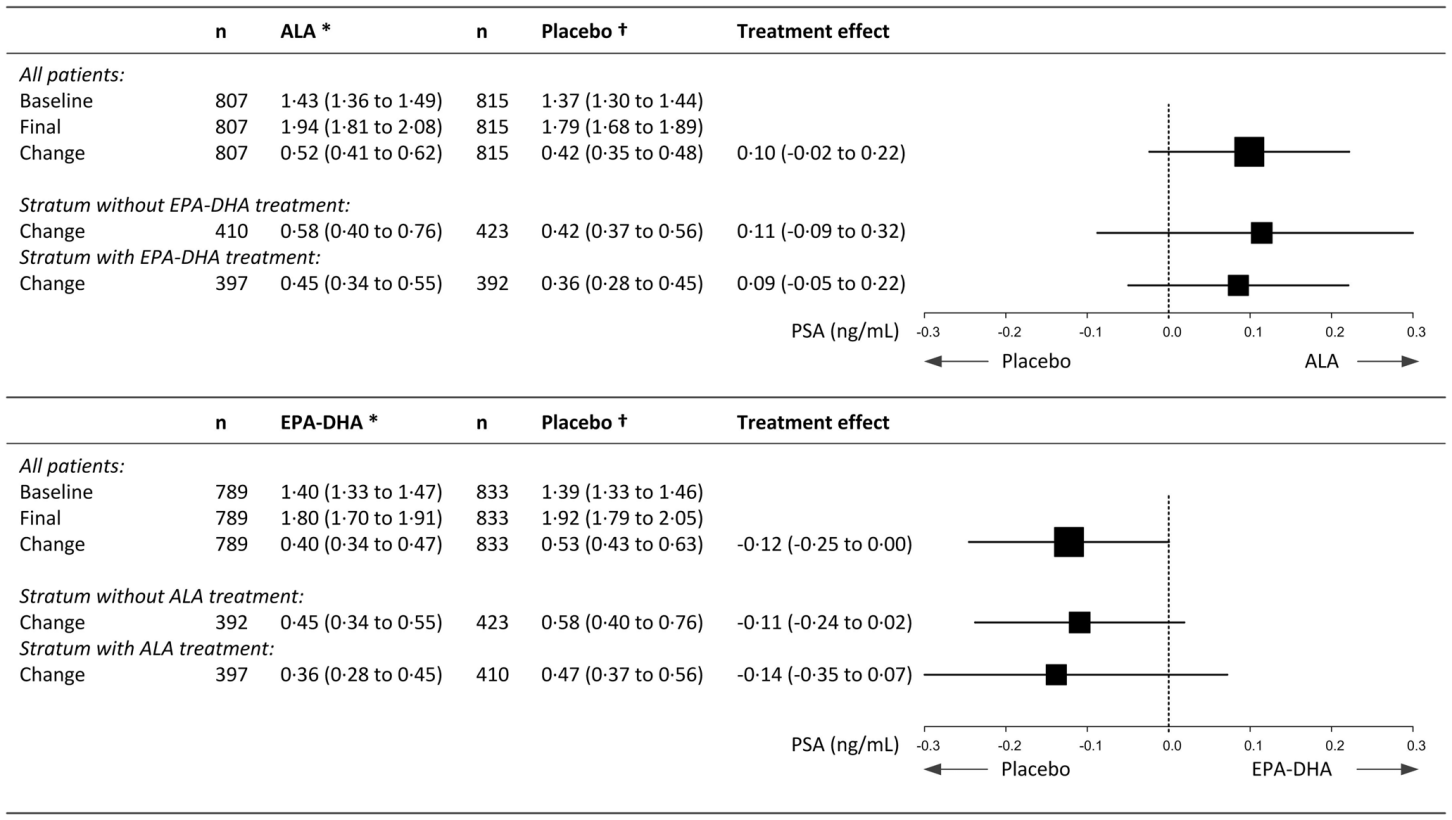
Effect of ALA supplementation on prostate specific antigen (PSA) concentrations (ng/mL) of 1622 male patients. Values are means (95% confidence interval). ALA = alpha linolenic acid, EPA = eicosapentanoic acid = EPA, DHA = docosahexanoic acid. For the two comparisons of ALA vs. placebo the test of heterogeneity was non-significant (Q-value 0.054; I-squared 0.000; P = 0.82), similar to the comparisons of EPA-DHA vs. placebo (Q-value 0.052; I-squared 0.000; P = 0.82).

Mean serum PSA increased by 0.52 (95% CI: 0.41 to 0.62) ng/mL in the combined ALA group compared to 0.42 (0.35 to 0.48) ng/mL in the combined placebo group during the intervention, an increase of 0.10 (−0.02 to 0.22) ng/mL (P = 0.12; Figure 3). There was no indication for an interaction between ALA and EPA/DHA For the two comparisons of ALA versus placebo the test of heterogeneity was non-significant (Q-value 0.054; I-squared 0.000; P = 0.82), similar to the comparisons of EPA-DHA vs. placebo (Q-value 0.052; I-squared 0.000; P = 0.82). A similar increase (0.11; −0.09 to 0.32 ng/mL) as in the combined groups was obtained for the patients in the group that received only ALA compared with the pure placebo group. Receiving EPA/DHA decreased mean serum PSA by 0.12 (−0.25 to 0.00) ng/mL compared with receiving placebo (Figure 3).

The risk of changing from a PSA <4 ng/mL to a PSA >4 ng/mL was increased by 15% (odds ratio 1.15; 95% CI 0.84 to 1.58) for ALA compared to placebo treatment (Table 2). Only one man with a baseline PSA value below 4 ng/mL was diagnosed with prostate cancer during the 40 mo of follow-up. His PSA value exceeded 4 ng/mL at the end of the study. We combined his data with those of 173 patients who also reached a PSA level above 4 ng/mL during the trial. ALA supplementation increased PSA velocity, but the 95% confidence intervals of the odds ratios in different categories of PSA velocity included 1.

**Table 2:** Risk of reaching a serum PSA concentration >4 ng/mL or an increased PSA-velocity after 40 months of ALA supplementation.

	ALA*	Placebo†	Odds ratio (95% CI)	P-value‡
	N = 807	N = 815		
**Final PSA >4 ng/mL**	92	82	1·15 (0·84–1·58)	0·38
**PSA Velocity**				
**>0·50 ng/mL per year**	68	54	1·30 (0·90–1·88)	0·17
**>0·75 ng/mL per year**	40	26	1·58 (0·96–2·62)	0·07
**>1·00 ng/mL per year**	23	12	1·96 (0·97–3·97)	0·06

PSA = prostate specific antigen), PSA Velocity = change in serum PSA concentrations per year, ALA = alpha linolenic acid, EPA = eicosapentanoic acid = EPA, DHA = docosahexanoic acid.

* 410 subjects treated with alpha linolenic acid alone plus 397 subjects treated with alpha linolenic acid plus EPA/DHA.

†423 subjects treated with placebo alone plus 392 subjects treated with EPA/DHA alone.

‡P-value t-test (two-sided).

## Discussion

Supplementation with an additional amount of 2 gram ALA per day during 40 months increased serum PSA concentrations of older post-myocardial infarction patients by 0.10 ng/mL. However, the 95% confidence interval ranged from −0.02 to 0.22 mg/mL. Effects of ALA supplementation on PSA velocity and on the combined endpoint of prostate cancer and a PSA level above 4 ng/mL were in the direction of a raising effect, but the confidence intervals were wide and all included 1.

To our knowledge, this is the first large-scale clinical trial that investigated the effect of ALA on serum PSA. The major limitation of our study was that we did not have enough prostate cancer cases to test the hypothesis that ALA supplementation increases the risk of this clinical outcome. It is important to realize that an effect on PSA cannot be translated one to one into an effect on prostate cancer. Serum PSA is a sensitive predictor of prostate cancer [16] but it is also increased in men with prostatitis and benign prostatic hyperplasia because PSA reflects prostate tissue volume. PSA levels >4.0 ng/mL do not always indicate a higher risk of cancer; only 25–33 percent of men with PSA >4.0 ng/mL indeed have prostate cancer. [17].

Another limitation is that we tested the hypothesis that ALA supplementation increases the serum PSA level in older patients who have had a myocardial infarction. This limits the generalization of the results of our trial to the general older male population or to younger men. We excluded men who died during the trial, who had prostate cancer or a PSA level above 4 at baseline, who refused final examination, or for whom we had insufficient blood (see figure 1). On average the patients in our sample were therefore slightly healthier than the full cohort. However, we do not expect that had a major impact on our outcomes because treatment groups were quite similar at baseline (Table 1). Thus, we consider bias from differential prognosis in the four groups unlikely. Strengths of the trial were that the compliance to ALA supplementation was excellent and the additional amount of 2 gram ALA per day was realistic and of the same size as the recommended dietary allowance. Also the follow-up of 40 months was longer than in other trials and allowed us to study the long-term effects of ALA supplementation on serum PSA.

Our results should be very carefully interpreted as the observed 95% confidence interval ranges from −0.02 to 0.22 ng/mL. This range includes zero, i.e. no effect. However, it also includes clinically meaningful effects. In the Copenhagen City Heart Study [13], men aged >60 yrs had a 10-year prostate cancer risk of approximately 1.1%, if their PSA between 0.01 and 1.00 ng/mL and the risk was 3.6% for men with a PSA between 1.01 and 2.00 ng/mL. Thus, in this range, a 1 ng/mL higher PSA level was associated with a 2.5% higher absolute 10-year prostate cancer incidence risk. Extrapolation from the Copenhagen City Heart Study [13] suggests that the observed mean increase in PSA of 0.10 ng/mL could lead to one extra new case of prostate cancer per 400 older men in 10 years. The upper limit of the confidence interval was 0.22 ng/mL, and such a rise could lead to one extra case per 180 men. When millions of men eat foods high in ALA they could easily reach the 2 g of ALA/d supplemented in our trial, and then the numbers start to add up. This reasoning is, however, highly speculative because it assumes a causal relation between the ALA-induced rise of PSA and prostate cancer incidence.

As far as we know we are the first to investigate the effect of pure alpha-linolenic acid by itself on PSA in men without prostate cancer. However, an earlier small study investigated the effect of flaxseed supplementation on prostate cancer proliferation rates and PSA in men with prostate cancer. [18] Flaxseed is a rich source of alpha-linolenic acid. Although patients with prostate cancer are quite different from our population and the study was small it should be noted that they did not show an effect on PSA and even showed a reduction in prostate cancer proliferation rates. [18].

The average dietary intake of ALA is approximately 2 g per day in men aged 50 to 70 yrs in The Netherlands. [19] In our trial we provided another 2 g which increased the total intake to about 4 g/d. Habitual intakes of 4 grams or more per day are rare, [19] but supplements rich in ALA can easily increase intake by 2 to 3 g/d, especially if people take teaspoons of flaxseed oil (linseed oil) daily. We observed a mean increase of 0.1 ng/mL in serum PSA concentrations after 40 months of supplementation with 2 g ALA per day, but confidence interval were too wide for definitive conclusions. Therefore, more trials are needed to investigate the effects of additional doses of 2–4 gram ALA per day.

The present study suggests that an additional amount of 2 g ALA per day may increase serum PSA, but it is unclear if and how ALA could influence prostate carcinogenesis. Sparse evidence from animal and cell structure studies suggests that ALA might act through other pathways than the very-long chain omega-3 fatty acids from fish fatty acids (eicosapentanoic acid and docosahexanoic acid). These have been claimed to be protective in the multistep process of carcinogenesis. [20], [21] Our study did not have the objective to investigate the effect of these longer chain n-3 fatty acids. However, the effect of the EPA/DHA group versus placebo was in the direction of a protective effect (−0.12; 95% CI −0.25 to 0.00 ng/mL). Two studies that investigated effects of intervention with the longer chain n-3 fatty acids found either no effect [22] or a protective effect [23]. In contrast, a large prospective case cohort study indicates an increased prostate cancer risk for men with high blood concentrations of longer chain n-3 fatty acids [24]. Therefore, effects of longer n-3 fatty acids on prostate cancer risk remain unclear.

In conclusion, an additional amount of 2 g ALA per day did increase the PSA concentration by 0.1 ng/mL, but the confidence interval ranged from a nil finding to a clinically meaningful effect. More research is needed to find out whether ALA influences the serum PSA level and the risk of prostate cancer and which levels of ALA intake are optimal for human health.

## Supporting Information

Protocol S1 **Trial protocol.**
(PDF)

Checklist S1 **CONSORT checklist.**
(DOC)
