# Supplementary material for: Effect of Alpha Linolenic Acid Supplementation on Serum Prostate Specific Antigen (PSA): Results from the Alpha Omega Trial
Source: PLoS One. 2013 Dec 11;8(12):e81519. doi: 10.1371/journal.pone.0081519 (PMC3859488; doi:10.1371/journal.pone.0081519)
Supplement: Protocol S1 — Trial protocol. (PDF) [file pone.0081519.s001.pdf]

ALPHA OMEGA TRIAL - RESEARCH PLAN

A. Specific aims

The Alpha Omega Trial is a randomized, placebo-controlled, double-blind dietary intervention study in 4000 postmyocardial infarction patients in The Netherlands to examine whether mortality from coronary heart disease (CHD) during 3 years of follow-up can be prevented by low doses of (very-)long-chain n-3 polyunsaturated fatty acids. The key objectives are:
1) to examine the effect of low-dose supplementation (400 mg daily) of eicosapentaenoic acid (EPA) and docosahexaenoic acid (DHA) on coronary mortality
2) to examine the effect of low-dose supplementation (2 g daily) of alpha-linolenic acid (ALA) on coronary mortality
The Alpha Omega Trial is the first food-based intervention study in which low doses of n-3 fatty acids (i.e. intake within the normal dietary range) are studied in relation to CHD. The trial started in May 2002 and 1849 subjects (46% of total study population) have now been enrolled (as of October 21, 2004). During this period, 53 deaths and 96 drop-outs have been recorded. Trials of ALA and EPA-DHA are still feasible in the Netherlands because n-3 enriched foods are not (yet) on the market and dietary supplements are not widely consumed. Furthermore, Dutch people have a high habitual intake of margarine on bread, which can be used as a vehicle for n-3 fatty acids to achieve double-blinding and good compliance. The Netherlands is a small country with a high population density and an efficient infrastructure, which facilitates the conduct of outpatient dietary intervention studies with complicated logistics. The trial is a joint initiative of the Division of Human Nutrition at Wageningen University, the National Institute for Public Health and the Environment (RIVM, Bilthoven) and the Netherlands Heart Foundation (The Hague). Patients are recruited by Dutch cardiologists. These collaborations facilitate the implementation of trial outcomes both in the clinic and in the population.

Cardiovascular diseases (CVD) are the most important cause of morbidity and mortality in the USA and other Western countries, and the prevalence of CVD is likely to increase in the future (Murray 1997). Despite efficient in-hospital interventions, the majority of patients do not benefit from emergency care. Over 60% of coronary deaths occur before patients reach the hospital and among hospitalized patients case-fatality is high (50% within 24 hours). Prevention is therefore of paramount importance, especially for sudden death. CVD also form an economic burden. Health care costs constituted 8.2% of the Dutch gross national product in 2000 (33 billion €), of which 13% in men and 10% in women was due to CVD. In the USA, even 17% of the health care budget was due to CVD in 1995, with annual costs exceeding $130 billion (Hodgson 1999). The potential impact of primary prevention with n-3 fatty acids is substantial in the Netherlands and other Western countries where intake of fish and ALA-rich foods is low. Prospective epidemiological studies (as discussed in section B: Background and Significance) have shown that modest increases in n-3 fatty acid intake could reduce CHD mortality by at least 25%. For the Dutch population aged 60-80 years (as studied in Alpha Omega Trial), this would reduce the number of deaths during 10 years of follow-up by 13,000, which is a relative reduction of 14% in this age category (modeling by Dr. Rudolf Hoogenveen, National Institute for Public Health and the Environment, Bilthoven, The Netherlands).

Sustainable changes in diet and lifestyle form the cornerstone of CVD prevention. Dietary advice, however, comes in many forms and the range of recommendations is broad, whereas the scientific foundation is often ambiguous and of variable quality. The Alpha Omega Trial aims at establishing the potentially large effect of dietary n-3 fatty acids in secondary and (indirectly) primary prevention of CHD. Findings from the Alpha Omega Trial will be communicated to the public, medical doctors, other health care providers and policy makers to integrate this knowledge in daily practice. Should a cardioprotective effect be found, attempts will be made to increase the intake of n-3 fatty acids in the Dutch population through dietary guidelines and preventive strategies. Different recommendations may be needed for EPA-DHA and ALA if distinct effects are found. There are many possibilities to increase intake of n-3 fatty acids. Oily fish species such as mackerel and salmon are important sources of EPA-DHA. Apart from consumption of fish and seafood products, fish oil or algae oil can be used for fortification of food products, either directly or through animal feed. By adding fish oil to margarine or other foods, people who dislike fish can also increase their EPA-DHA intake without a need to take fish oil capsules. Dietary recommendations for ALA could focus on vegetables or vegetable oils, nuts, ALA supplements, or ALA-enriched foods.

B. Background and Significance

Whether dietary n-3 polyunsaturated fatty acids are causally related to CHD is an important, unresolved question in preventive cardiology. Essential n-3 fatty acids are eicosapentaenoic acid (EPA; C20:5,n-3) and docosahexaenoic acid (DHA; C22:6,n-3) on one hand, and their parent compound alpha-linolenic acid (ALA; C18:3,n-3) on the other hand (Figure 1). EPA and DHA are almost exclusively derived from fatty fish, such as mackerel and salmon (Hepburn 1986, Kris-Etherton 2000). ALA is derived from various dietary sources, including soy and rapeseed oil, whole grain, vegetables and fruits, and meats (Voskuil 1996). The human body can convert ALA into EPA, but only to a very limited extent (Sinclair 2002), and adequate dietary intake of very-long-chain n-3 fatty acids is therefore crucial. The ratio of n-6 to n-3 fatty acids in the diet of Western populations is still much higher than that recommended (Kris-Etherton 2000).

Epidemiological studies by Kromhout *et al* suggested that weekly intake of 1-2 servings of fish, especially fatty fish, could reduce the risk of CHD by 30-50% (Kromhout 1985, Kromhout 1995, Oomen 2000). Prospective cohort studies in this field have recently been summarized in a meta-analysis by He *et al*, showing a decrease in CHD mortality by 15% for fish consumption once per week, by 23% for fish consumption 2-4 times per week and by 38% for fish consumption 5 or more times per week, compared to fish intake less than once per month (He 2004a).

Fish and n-3 fatty acids probably have a stronger effect on fatal than on nonfatal CHD (Mozaffarian 2003). In a recent nested case-control study conducted within the Cardiovascular Health Study, higher plasma phospholipid levels of EPA-DHA were associated with a lower risk of fatal CHD but not nonfatal myocardial infarction (Lemaitre 2003). Experimental research brought about the intriguing hypothesis that very-long-chain n-3 fatty acids could prevent cardiac arrhythmia (Kang 1996), which precedes cardiac arrest. In line with this hypothesis, a decreased risk of sudden cardiac death was found in participants in the US Physicians' Health Study who consumed fish at least once a week (Albert 1998).

Figure 1. Simplified metabolic pathway of n-6 and n-3 polyunsaturated fatty acids

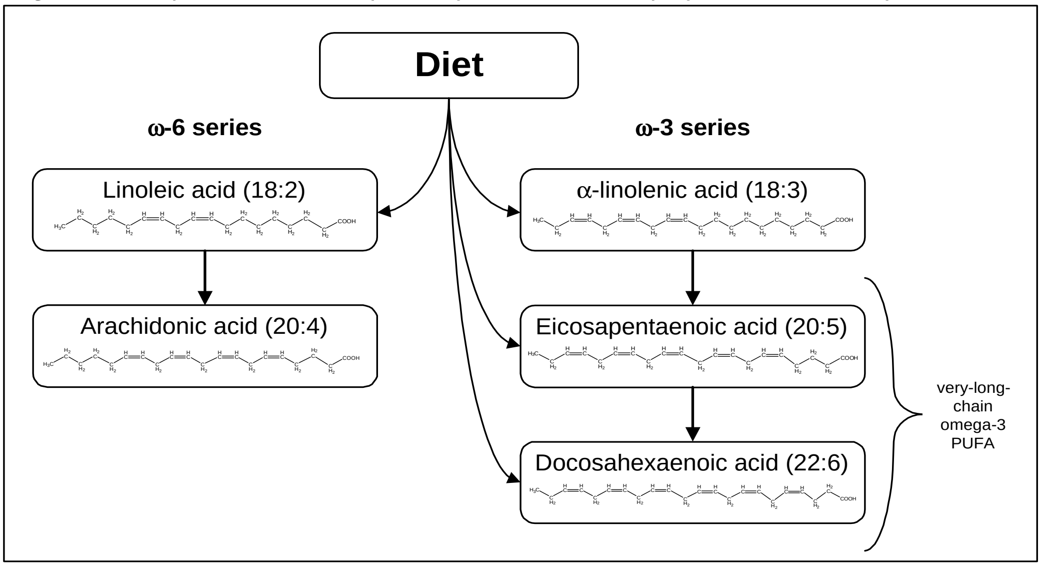

Siscovick *et al*, in a case-control study, observed an inverse dose-response relationship of both dietary intake and erythrocyte membrane levels of very-long-chain n-3 fatty acids with primary cardiac arrest (Siscovick 1995). In general, stronger associations between n-3 fatty acids and cardiovascular endpoints are found for biomarkers (fatty acids in plasma phospholipids, erythrocytes or adipose tissue) than for dietary questionnaire data (e.g. Erkkilä 2003). In the Physicians' Health Study, the relative risk of sudden death was dramatically reduced by 81% in men whose blood levels of long-chain n-3 fatty acids were in the fourth quartile compared to risk for men in the lowest quartile (Albert 2002). Part of the cardioprotective effect of EPA-DHA, however, may be counteracted by mercury in fish as shown in the EURAMIC study (Guallar 2002).

The link between dietary EPA-DHA and cerebrovascular disease is less established than for CHD. In the Zutphen Study, weekly fish consumption was associated with a 51% reduction in risk of stroke (Keli 1994). Prospective cohort studies in this field have recently been summarized in a meta-analysis by He *et al*, showing a decrease in risk of stroke by 13% for fish consumption once per week, by 18% for fish consumption 2-4 times per week and by 31% for fish consumption 5 or more times per week, compared to fish intake less than once per month (He 2004b).

An overview of trials of fish or n-3 fatty acids is given in Table 1. An inverse association of fatty fish consumption with coronary mortality was found in the DART-trial (Burr 1989), a dietary intervention study in over 2000 subjects who had recently suffered a myocardial infarction. The risk of total and coronary mortality in this trial was reduced by 30% in those subjects who had been advised to consume 2-3 fatty fish meals per week or (if subjects were reluctant to eat fish) equivalent doses of fish oil in capsules. These findings strongly support a protective effect of EPA-DHA against CHD. Risk reductions were similar in fish eaters and in subjects who took the fish oil capsules, suggesting that it is indeed EPA-DHA intake that accounts for the protective effect. It should be noted, however, that fish advice did not reduce coronary risk when the DART trial was repeated in patients with angina pectoris (Burr 2003). To further test the hypothesis, the open-label GISSI-trial was conducted in over 11,000 subjects who were enrolled within 3 months after an acute myocardial infarction. Moderate doses of EPA-DHA in fish oil capsules were prescribed for 3-5

years (GISSI-Prevenzione Investigators 1999). With a compliance of 70%, the intake of EPA-DHA was effectively increased by ~600 mg/d. Subjects who received fish oil capsules had a 20-35% reduction in coronary mortality, a 14-20% reduction in all-causes mortality and even a 26-45% reduction in sudden cardiac death. Fish oil supplementation (1.8 g EPA-DHA daily for 2 years) was recently studied in survivors of ventricular tachyarrhythmias, but in contrast to expectations no antiarrhythmic effect was found (Raitt 2003).

Evidence for the parent compound ALA is suggestive, but less consistent. A recent meta-analysis of 5 prospective cohort studies, including the Zutphen Elderly Study (Oomen 2001), showed an inverse relationship between ALA intake and risk of fatal CHD, which was borderline statistically significant (relative risk of 0.79, 95% confidence interval 0.60-1.04) (Brouwer 2004). In the EURAMIC Study the concentration of ALA in adipose tissue was significantly inversely related to incident myocardial infarction, with a relative risk of 0.42 in the upper *vs.* lower quintile (Guallar 1999). However, the relationship was attenuated after adjustment for smoking. Recently, plasma ALA has been inversely associated with fatal CHD (Lemaitre 2003) and overall mortality (Erkkilä 2003). Trials of ALA and CHD are lacking. In the Lyon Diet Heart Study, 605 subjects who suffered a myocardial infarction consumed a Mediterranean diet with an ALA-enriched margarine for 2 years (De Lorgeril 1999). The trial was terminated prematurely when a 70% reduction in the reinfarction rate and mortality was achieved. Apart from increased intake in ALA, participants in the Lyon Diet Heart Study ate more fish, fruits, and vegetables, which may have contributed to the substantial decrease in coronary risk. Thus, data are suggestive for a cardioprotective effect of ALA, but its causal role still needs to be established in large intervention studies (Kris-Etherton 2002). With regard to cerebrovascular disease, data on the effect of ALA intake are scanty. Blood ALA levels were inversely related to incident stroke in MRFIT (Simon 1995). However, no reduction in stroke was found in the Lyon Diet Heart Study (De Lorgeril 1999).

Table 1. Randomized trials of n-3 fatty acid supplementation in coronary subjects

**Study**	GISSI<br>(GISSI Group 1999)	Lyon Diet Heart Study,<br>(De Lorgeril 1999)	DART<br>(Burr 1989)
**Blinding**	Open-label	Single-blind	Single-blind
**No. of subjects**	11,324 M/F	605 M/F	2,033 M
**Date of MI**	<3 months	~6 months (recovered)	~41 days
**Age**	No age limit	<70 years	<70 years
**Follow-up**	3.5 (range: 3-5) years	3.8 years	2 years
**Intervention**	➢ 885 mg EPA-DHA<br>➢ 885 mg EPA-DHA + 300 mg vitamin E<br>➢ 300 mg vitamin E<br>➢ Placebo	➢ Mediterranean diet (↑oleic acid, ↑ALA)<br>➢ Control diet	Dietary advice:<br>➢ Fat (<30 En%, P/S=1)<br>➢ Fish (200-400 mg/wk)<br>➢ Fiber (18 g/d)<br>➢ Fat + fish<br>➢ Fat + fiber<br>➢ Fish + fiber<br>➢ Fat + fish + fiber<br>➢ No advice
**Results**	$RR_{EPA\text{-}DHA}$<br>-All-cause mortality: 0.80<br>-Coronary mortality: 0.65	$RR_{Mediterranean\ diet}$<br>-Cardiac mortality + non-fatal MI: 0.28	RR_{fish}<br>-All-cause mortality: 0.73<br>-Coronary mortality: 0.68

In conclusion, the DART-trial and the Lyon Diet Heart Study, although randomized, do not provide a solid basis for dietary recommendations on n-3 fatty acids because these studies were not blinded. Also for the GISSI-trial, the open-label design and the fact that subjects were still in the acute phase after myocardial infarction hamper inference drawing. The American Heart Association emphasized the need for additional studies with cardiovascular end points that go beyond the measurement of surrogate CVD markers to evaluate the effects of fatty acids in humans (Kris-Etherton 2001). About the AHA's recent guideline for fish oil supplements, Dr. Scott Grundy stated that *'...Available evidence is suggestive of benefit in the immediate post-MI period, but a solid recommendation cannot be made without more definitive controlled clinical trials.*' (Grundy 2003). In June 2004, NIH organized a Working Group Meeting to discuss a comprehensive report on n-3 fatty acids and CVD by the Agency for Healthcare Research and Quality, Rockville, MD (*Effects of Omega-3 Fatty Acids on Cardiovascular Disease*. AHRQ Publication No. 04-E009-2, March 2004). The authors of this report concluded that *'...Well-designed, multi-center RCTs are needed to assess the effect of omega-3 fatty acid consumption on CVD outcomes in primary and secondary prevention settings. The trial design should include a period of long-term follow-up for 3 to 5 years so that long-term effects of omega-3 fatty acids can be monitored...'* and also that *'...The potential effect of ALA is unknown. Current data sets are of poor quality and are too limited for adequate assessment. More trials are needed to confirm or report the effect of ALA, separate from fish or fish oil, on CVD outcomes....The relative effect of ALA versus fish oil is not well defined. Comparative trials between these 2 supplements should be conducted...'* The Alpha Omega Trial could make a major contribution to evidence-based nutrition by filling in this knowledge gap.

C. Preliminary Studies

C1. Previous research on fish and n-3 fatty acids

Fatty acids are not only important in relation to lipid metabolism but may also directly affect CHD by influencing ventricular fibrillation. In 1985, Kromhout *et al* published a paper in the *New England Journal of Medicine* on the inverse relation between fish consumption and 20-year coronary heart disease mortality in the Zutphen Study (Kromhout 1985). This paper evolved from the so-called Inuit (Eskimo) story. A comparative study between Inuits and Danes showed that CHD mortality among Inuits was only one tenth of the mortality among Danes (Bang 1985). It was hypothesized that this was due to the very high consumption of seafood by the Inuits, i.e. 400 grams per day. Seafood is a rich source of the very-long-chain n-3 fatty acids EPA and DHA.

The Zutphen Study was the first to show a protective effect of a small amount of fish on CHD mortality. Men who consumed fish once or twice a week had a 50% lower risk compared with men who did not consume fish (Kromhout 1985). In the Zutphen Study also an inverse relation was observed between fish consumption and 15-year incidence of stroke (Keli 1994). The protective effect of a small amount of fish on CHD mortality was confirmed in a study of a Dutch general practitioner who followed 272 of his subjects over 64 years of age for 17 years (Kromhout 1995). An analysis of the dietary data of the Dutch, Finnish and Italian cohorts of the Seven Countries Study showed that the protective effect was most pronounced for fatty fish

(Oomen 2000). Fatty fish is a rich source of EPA and DHA and it is therefore very likely that the protective effect of fish against CHD can be attributed to these n-3 fatty acids. Evidence on fish consumption, EPA and DHA and cardiac death was summarized in an editorial for the *Journal of the American Medical Association* (Kromhout 1998) and a review in *Circulation* (Kromhout 2002).

In 1986 we carried out a small comparative study of long-term fish eaters and controls within the Zutphen Study. The difference in fish consumption between the two groups was 30 grams per day (~1 serving of fish per week) during 26 years. A 25% difference was shown in serum triglyceride level between the two groups (Kromhout 1996). This amounted even to 40% for the triglyceride concentration in the very atherogenic Intermediate Density Lipoprotein (IDL) fraction, There was no difference between the two groups in platelet function and major eicosanoid metabolites in blood. The habitual fish consumers had, however, substantially higher levels of EPA and DHA in the phospholipid fraction in serum compared with controls (Van Houwelingen 1989). This suggests an effect of fish consumption on the composition of membranes, which may play a role in the prevention of conduction disturbances and ventricular fibrillation.

Recently, the effect of alpha-linolenic acid (ALA) on CHD has been examined in the Zutphen Study (Oomen 2001). In contrast to expectation the intake of ALA was positively associated with 10-year incidence of CHD. ALA intake, however, appeared to be strongly correlated with the intake of *trans* fatty acids. After adjustment for *trans* fatty acids there was no relationship between ALA intake and risk of CHD. This is in contrast with several other observational studies that showed a protective effect of ALA on incident CHD, as summarized in a recent meta-analysis (Brouwer 2004).

To examine the preventive potential of low doses of EPA-DHA and ALA in CHD, researchers at Wageningen University and the National Institute for Public Health and the Environment (RIVM) initiated the Alpha Omega Trial on commission of the Netherlands Heart Foundation (topdown program 2000T401). The trial started in May 2002 and a pilot study was completed in March 2003 (see section C2). The Division of Human Nutrition, Wageningen University (www.ftns.wau.nl/nutepi; director: Dr. Frans J. Kok), has large expertise in nutritional research, including controlled feeding experiments and dietary intervention studies in free-living individuals. The RIVM has coordinated a number of large cohort studies, including the Zutphen Study and MORGEN Project. Also, the RIVM is involved in international population-based studies, such as the Seven Countries Study, the FINE Study and the HALE project that are coordinated by Dr. Daan Kromhout. Long-term fruitful collaboration exists between RIVM and Wageningen University. Dr. Daan Kromhout is currently Director of the Nutrition and Consumer Safety Division at RIVM and Professor of Public Health Research at Wageningen University. He is the initiator and Principal Investigator of the Alpha Omega Trial.

C2. Alpha Omega Trial – Pilot Study

Aim and design

The Alpha Omega Trial is a multicenter study of n-3 fatty acids and coronary mortality in 4000 postmyocardial infarction patients that started in May 2002. A pilot study was set up in 5 Dutch clinics 1) to test study procedures and assess the feasibility of the trial, 2) to assess compliance, and 3) to examine whether intervention with n-3 fatty acids would cause adverse effects on health. The pilot study included 400 subjects

who were randomized between May-October 2002. Randomization took place after a 4-6 week run-in on placebo margarine. Intervention comprised daily use of 20 g of trial margarine containing 1) 400 mg EPA-DHA, 2) 2 g ALA, 3) 400 mg EPA-DHA & 2 g ALA, or 4) placebo, following a 2x2 factorial design. Pilot participants were examined at baseline and after 3 months of intervention (mean: 13.1 wk) by trained research nurses. Pilot data were analyzed by the Data and Safety Monitoring Board (Dr. Eric Boersma, independent biostatistician; Thorax Center, Erasmus Medical Center Rotterdam) in strata of treatment, with blinding towards type of treatment.

Patient recruitment

Patient recruitment was performed by cardiologists of 5 clinics in different regions in the Netherlands. Patients from hospital ‘Leyenburg’, The Hague, were examined at home because insufficient facilities were available at the clinic. Patients from other hospitals visited the outpatient clinic for examination. The regional distribution of 400 pilot participants in the Netherlands was 53% for The Hague, 24% for Zwolle, 10% for Goes and 14% for Eindhoven. A response rate of 30% was achieved, thus around 1300 eligible subjects were contacted to yield 400 pilot participants. Among subjects aged 60-80 years old who fulfilled the inclusion criteria of a myocardial infarction, the most important reasons for non-participation could be roughly classified in three categories namely 1) feeling too old to fulfill the demands of participation, 2) too much difficulties with filling out questionnaires, and 3) spending time abroad, which complicates use of trial margarine.

Table 2. Baseline characteristics of participants: pilot study of Alpha Omega Trial (*n*=400)

Age (yr)	68.8 ± 5.6
Women	25.0
Belonging to ethnic minority	1.3
Education (highest achieved level):	
-Primary school	22.7
-Secondary/vocational	69.0
-Higher vocational/university	8.4
Living alone	17.7
Alcohol use (>1 glass/wk)	61.9
Current smoking	23.3
Good health status (self-reported)	75.5
Medical history (self reported):	
-Hypertension	45.7
-Diabetes	13.6
-Cancer	11.4
-Angina pectoris	17.9
-Heart failure	27.0
-Claudicatio intermittens	11.6
-Stroke	6.8
-Aneurysm of abdominal aorta	4.5
Height (cm)	171.4 ± 8.4
Body weight (kg)	81.7 ± 12.4
Body mass index (kg/m^2)	27.8 ± 3.8
Blood pressure (mmHg):	
-Systolic	142.3 ± 21.0
-Diastolic	81.4 ± 11.0
Heart rate (bpm)	69.9 ± 11.9
Blood lipids (mmol/L):	
-Total cholesterol	5.2 ±1.0
-HDL cholesterol	1.3 ±0.4
-Triglycerides	2.0 ±1.1
Plasma glucose	6.0 ±1.9

Values are mean ± SD or percentage

Characteristics of pilot cohort

The pilot cohort comprised 300 men and 100 women aged 60-80 years (mean: 69 years) with a documented history of myocardial infarction up to 10 years before randomization (Table 2). The prevalence of preexisting cardiovascular diseases and risk factors was high, as expected. Characteristics in men and women did not markedly differ except that men had a higher alcohol intake (63.9% in men *vs.* 54.0% in women with habitual intake >1 glass/wk) and were higher educated (9.7% in men *vs.* 4.0% in women with higher vocational or university education). Randomization yielded a balanced distribution of subjects over the treatment groups (24%, 25%, 27% and 24%). Subject characteristics were also balanced over the groups, as shown in Table 3.

Table 3. Effect of randomization on subject characteristics in the pilot study (*n*=400)

Variable	Treatment group in random order (blinded analysis)				P-value*
	A	B	C	D	
Distribution of subjects (%)	23.5	25.3	27.3	24.0	0.88
Age (y), mean ± SD	69.9 ± 5.7	68.8 ± 5.4	69.2 ± 5.8	69.7 ± 5.3	0.60
Gender (women, %)	24.7	19.0	25.9	31.6	0.25
Smoking (current, %)	20.4	12.0	15.7	11.6	0.49
Education (low, %)	34.4	38.0	38.9	28.4	0.51
Marital status (living alone, %)	21.5	17.0	18.5	13.7	0.56
Alcohol use (>1 glass/wk, %)	62.4	64.0	58.3	63.2	0.84

* Two-sided P-value, obtained by ANOVA (for age) or Chi-square test (other variables)

Trial margarines

Margarine was chosen as the vehicle for increasing the intake of n-3 fatty acids in the Dutch population because the habitual intake of margarine (mainly on bread) is high in the Netherlands. Four types of trial margarines were successfully produced by Unilever, Vlaardingen, The Netherlands. Trial margarines either contained [1] 2 g alpha-linolenic acid (ALA; C18:3n-3) per 20 g, [2] 400 mg eicosapentaenoic and docosahexaenoic acid (EPA-DHA; C20:5n-3/C22:6n-3) per 20 g, [3] 2 g ALA and 400 mg EPA-DHA per 20 g, or [4] placebo (oleic acid). Testing in two independent laboratories showed that target levels for fatty acid composition of trial margarines were matched very closely and that deviations among batches either at the same or at different production days were negligible. An independent taste panel concluded that the margarines had a minimal closed shelflife of 12 weeks and an open shelflife of 2 weeks, but also after 16 weeks 'fish oil' perception remained below the threshold value. There were no differences in taste, appearance and concentration of linoleic acid among the four types of trial margarines. Blinding of research staff and participants towards type of treatment was achieved and maintained. At the end of the 3-month intervention, 87% of the subjects indicated that they did not know which treatment group they were in. Of those who had an opinion, 2% believed they were allocated to EPA-DHA, 5% to ALA, 3% to the combined treatment and 2% to placebo (P=0.15 for differences among groups).

Margarines were stored in a freezing house, under strictly controlled and hygienic conditions (Vrieshuis Vink, Beverwijk, The Netherlands). Packages containing 8 tubs (250 g each) of trial margarine were successfully delivered every 12 weeks at patients' home by courier service (Valid Express, Amsterdam; www.validexpress.nl), under supervision of a specialized logistics company (LOF Logistiek, Egmond aan den Hoef, The Netherlands; www.loflogistiek.nl). No major problems were encountered during storage or cooled transport. During the pilot phase, 89% of the participants had received a first delivery of margarine, 56% a second delivery, 20% a third delivery, and <1% (only the first randomized participant) a fourth delivery. In total, there were around 1,250 margarine deliveries during the pilot phase.

Blood sampling procedure

Blood was drawn in fasting condition (>8 hours) in 43% of the subjects, and in non-fasting condition in the remainder of the subjects. Blood samples that were collected

at clinical research sites were successfully delivered by standard postal service to the central laboratory (National Institute for Public Health and the Environment [RIVM], Bilthoven, the Netherlands). Of all baseline blood samples, 89% was delivered at RIVM within 24 hours and 96% within 48 hours. This is in accordance with the mean of 95% and above the minimum of 85%, which is guaranteed by the Dutch postal service (TPG-Post, The Hague, the Netherlands).

The blood handling procedure for the Alpha Omega Trial was extensively tested in 20 healthy volunteers before the start of the trial (Giltay 2003, article included in Appendix). Blood was sampled from subjects in non-fasting condition and part of it was directly processed and stored at –80 °C. The remainder was sent by postal mail and processed after a delay of 24 and 48 hrs, during which it had been stored at room temperature. After 48-hrs delay, subjects could still be adequately ranked for blood glucose, lipids, C-reactive protein, and different fatty acids (including EPA and DHA in plasma cholesteryl esters). The variability (coefficient of variation (CV)) and reliability of n-3 fatty acids in cholesteryl esters after 24 hrs and 48 hrs compared with direct processing were calculated. The CV for EPA and DHA in EDTA-plasma cholesteryl esters were 5.4% and 1.3%, respectively, after 1 day, and 8.1% and 8.4%, respectively, after 2 days. The reliability coefficients for EPA and DHA were >0.95 at both days (P<0.0005), reflecting high stability of these fatty acids. Similarly for glucose, lipids, and C-reactive protein (in plasma or serum) the CV for the paired difference was small (≤ 3.2%) and the reliability was high (≥ 0.97) at day 1 *vs.* day 0. A delay of 2 days yielded a somewhat larger CV (≤ 4.9%) and lower reliability (≥ 0.90). Mailing blood samples enables the study of large numbers of subjects at different research sites. Blood specimens can also be collected at individuals' homes without the need for strict preanalytic procedures (i.e. direct centrifugation, separation, dispensing, and freezing). In the Alpha Omega Trial with 4000 patients and a central laboratory, this procedure is highly cost-effective.

Health effects

Two subjects died from heart failure during 3 months of follow-up and there were 7 drop-outs (1.8%). Reasons for drop-out were lung cancer with chemotherapy (*n*=1), urticaria and itching of the skin (*n=1*), unintended weight gain (*n=1*), stress-related issues (*n=2*), dislike of the trial margarine (*n=1*), and no specific reason (*n=1*). Changes in self-perceived health (rated on 3-point scale) during intervention did not differ among treatment groups (*P*=0.60). However, the number of hospital admissions was unequally distributed among the treatment groups (14.75%; 10.45%; 4.35% and 2.38%; *P*=0.022). The total number of hospitalizations was 24, of which 9 were related to CVD. For most hospitalizations (e.g., cataract, osteoporotic fracture, cancer) a link to recent intake of n-3 fatty acids was not plausible. Findings on hospital admissions were therefore attributed to chance as the most likely explanation and treatment codes remained sealed.

Baseline and 3-month blood lipids and glucose were analyzed using standard kits and an autoanalyzer (Hitachi 912, Roche Diagnostics, Basel, Switzerland). LDL cholesterol was calculated using the Friedewald formula. The intra and interassay coefficients of variations were 0.9% and 1.8% for glucose, 0.8% and 1.7% for total cholesterol, 1.3% and 2.6% for HDL and 1.5% and 1.8% for triglycerides, respectively. After 3 months of intervention, total and LDL cholesterol showed an overall reduction from 5.06±1.01 to 4.95±0.98 mmol/L, and from 2.88±0.92 to 2.72±0.90 mmol/L, respectively, but changes were not significantly different among the four treatment groups (Table 4). Serum triglycerides, HDL cholesterol and plasma

Table 4. Effect of treatment on plasma lipids and glucose: pilot study of Alpha Omega Trial (*n*=400)*

	Baseline	3 months	P-value
Total cholesterol (mmol/L)	4.94 (0.87)	4.86 (0.95)	
	5.17 (1.07)	5.13 (1.05)	
	5.24 (1.15)	5.15 (1.07)	
	4.95 (0.92)	4.74 (0.77)	0.50
HDL cholesterol (mmol/L)	1.27 (0.32)	1.25 (0.29)	
	1.29 (0.34)	1.26 (0.34)	
	1.18 (0.26)	1.20 (0.37)	
	1.26 (0.40)	1.27 (0.37)	0.71
LDL cholesterol (mmol/L)	3.04 (1.00)	2.96 (0.92)	
	2.75 (0.84)	2.57 (0.78)	
	2.94 (0.95)	2.87 (1.01)	
	2.87 (0.85)	2.63 (0.86)	0.19
Triglycerides (log-mmol/L)	0.62 (0.43)	0.63 (0.44)	
	0.57 (0.43)	0.65 (0.49)	
	0.56 (0.50)	0.65 (0.48)	
	0.63 (0.52)	0.62 (0.53)	0.38
Glucose (mmol/L)	6.48 (2.16)	6.44 (2.22)	
	5.79 (1.79)	6.08 (1.71)	
	6.21 (2.36)	6.12 (2.02)	
	5.95 (1.80)	6.28 (2.00)	0.45

* Mean (SD), with P-value for treatment effect; rows represent treatment groups, in random order

glucose remained relatively stable during intervention and changes from baseline were similar across treatments (Table 4). There was neither a treatment effect on systolic blood pressure (*P*=0.99), diastolic blood pressure (*P*=0.79), body weight (*P*=0.97) and body mass index (*P*=0.94) (data not in Table). Heart rate was significantly increased after 3 months (+3.1±9.0 bpm), but this change did not differ among treatment groups (*P*=0.56).

Compliance
In 8.6% cases of margarine deliveries one or more unopened tubs were returned to the courier (predominantly trial margarine, and not run-in margarine). If all 8 tubs of margarine are consumed during a 12-week-period, the average intake of margarine is 23 grams per day. Since only few unopened tubs were returned during the pilot phase, anticipated levels of n-3 intake are likely to be achieved. Table 5 shows effects of treatment on fatty acids in plasma cholesteryl esters, as a biomarker of compliance. There were strong increases in ALA in two groups (+35% and +29%; *P*=0.0001), whereas ALA decreased in two other groups (–10.4% and –22.0%). Similarly, there were strong increases in EPA in two groups (+40% and +33%; P=0.004) and small decreases (–1.6%, and –12.1%) in two other groups. Changes in cholesteryl fatty acid composition were confined to n-3 fatty acids (Table 5).

Conclusion
No major problems were encountered with regard to accrual of patients by cardiologists, data collection by research nurses, margarine distribution by courier service, data processing or other logistics. Double-blinding and good compliance were achieved and there were no major adverse effects on health. From the pilot study it can be concluded that the Alpha Omega Trial is safe and feasible.

Table 5. Effect of treatment on fatty acid composition of plasma cholesteryl esters: pilot study of Alpha Omega Trial (*n*=400)*

	Baseline (mass%)	3 months (mass%)	P-value
Saturated fatty acids	13.6 (0.8)	13.6 (1.3)	
	13.9 (1.0)	13.9 (0.4)	
	13.9 (0.9)	13.9 (0.9)	
	13.9 (0.8)	13.8 (0.9)	0.92
Monounsaturated fatty acids	19.9 (3.1)	20.5 (2.4)	
	20.0 (3.0)	19.5 (2.6)	
	21.5 (3.9)	20.2 (3.3)	
	21.5 (2.9)	20.3 (1.9)	0.11
Polyunsaturated fatty acids	62.1 (3.5)	63.9 (2.5)	
	62.5 (4.9)	64.4 (4.2)	
	63.6 (3.5)	64.7 (3.5)	
	63.8 (4.6)	63.5 (2.9)	0.15
n-6 polyunsaturated fatty acids	58.4 (4.2)	60.5 (3.2)	
	59.8 (5.1)	61.1 (4.2)	
	60.8 (3.9)	61.6 (3.8)	
	61.2 (4.8)	61.1 (3.1)	0.21
Linoleic acid	48.8 (4.4)	51.0 (3.7)	
	50.1 (4.9)	52.1 (4.6)	
	50.4 (4.7)	50.0 (2.9)	
	51.1 (4.4)	52.4 (4.1)	0.11
n-3 polyunsaturated fatty acids	2.38 (1.05)	2.26 (1.06)	
	2.49 (0.56)	3.06 (0.60)	
	2.59 (0.95)	2.89 (1.00)	
	3.45 (1.62)	3.11 (1.10)	0.014
Alpha-linolenic acid	0.48 (0.12)	0.43 (0.11)	
	0.50 (0.12)	0.39 (0.07)	
	0.55 (0.13)	0.74 (0.18)	
	0.55 (0.14)	0.71 (0.17)	0.0001
Eicosapentaenoic acid (log transformed)	0.021 (0.363)	0.356 (0.313)	
	0.075 (0.577)	0.358 (0.441)	
	-0.042 (0.492)	-0.058 (0.500)	
	0.433 (0.651)	0.304 (0.511)	0.0036
Docosahexaenoic acid (log transformed)	-0.349 (0.269)	-0.208 (0.230)	
	-0.353 (0.297)	-0.307 (0.144)	
	-0.464 (0.297)	-0.448 (0.363)	
	-0.208 (0.381)	-0.324 (0.382)	0.0063

* Mean (SD), with P-value for treatment effect; Rows represent treatment groups, in random order

D. Research Design and Methods

D1. Design

The Alpha Omega Trial has a parallel, 2x2 factorial design with random allocation of equal numbers of subjects to one of the four treatment groups:

I. 400 mg/d eicosapentaenoic acid and docosahexaenoic acid (EPA-DHA) + placebo for alpha-linolenic acid (ALA)
II. 2 g/d ALA + placebo for EPA-DHA
III. 400 mg/d EPA-DHA + 2 g/d ALA
IV. Placebo for EPA-DHA + placebo for ALA

The total intervention period is 3 years, proceeded by a 4-6 week run-in period on placebo margarine. Research staff, participants and couriers for margarine transport are blinded towards the type of margarine that subjects receive.

Figure 2. Design of Alpha Omega Trial

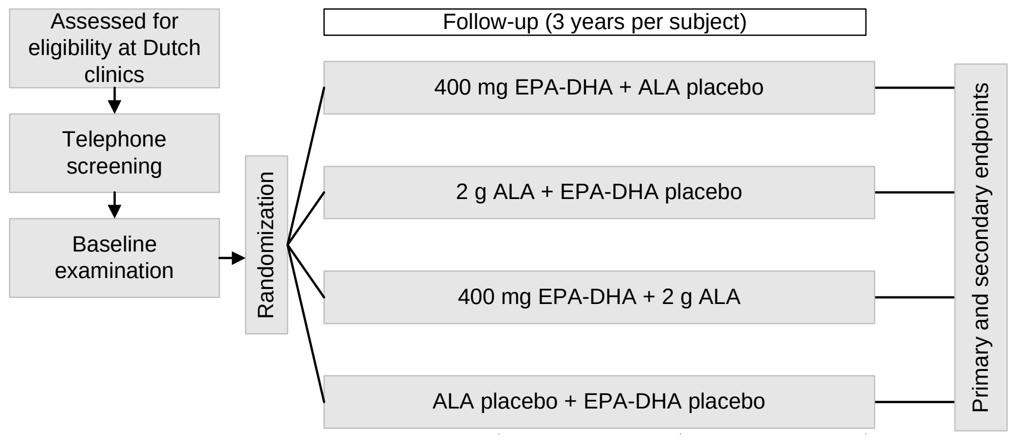

D2. Study population

Criteria for participation

A total of 4000 subjects are randomized. Eligible for participation are men and women aged 60-80 y who had a clinically diagnosed myocardial infarction up to 10 y before randomization. Subjects are excluded if they meet any of the following criteria:

1. Living in an institution (e.g. nursing home) with a central meal service
2. Recent (<4 weeks) or current participation in a scientific study
3. Habitual margarine intake <10 g per day
4. Habitual fish intake >150 g per day
5. Habitual alcohol intake >5 drinks per day
6. Recent (<2 weeks) use of fish oil capsules or other n-3 supplements
7. Presence of cancer with less than 1 year of life expectancy
8. Dementia or severe cognitive decline (MMSE score ≤ 21)
9. Unintended weight loss >5 kg in the past year
10. Severe heart failure (according to criteria of the European Society of Cardiology)
11. Unwilling or unable to comply with study procedures

Patient recruitment
In the Netherlands, approximately 255,000 men and women have a lifetime history of myocardial infarction, exclusive of silent infarctions (Netherlands Heart Foundation 1995). Around 195,000 of these subjects are aged 60-80 years. After taking into account the exclusion criteria, the size of the catchment population for the present trial can be reasonably estimated at 75,000 – 100,000 men and women. To include 4000 subjects, a response of 4-5% on a nation-wide basis is needed. Subjects are recruited in collaboration with Dutch cardiologists. Overall response (eligible patients that are contacted and eventually randomized) is around 25%. In October 2004, a total of 18 clinics were included in the trial and it is expected that this number is sufficient to include the remainder of the cohort (*n*=2151) before the end of 2005.

Generalizability
The population for the Alpha Omega Trial comprises older, free-living men and women who have a history of myocardial infarction. The background risk of coronary mortality strongly increases after a myocardial infarction, which makes it possible to conduct a trial as proposed in this application with a feasible number of subjects. The strength of the association between dietary parameters and coronary endpoints (i.e., relative risk) is not likely to be strongly influenced by background risk, as has been shown for classical risk factors (Verschuren 1995, Van den Hoogen 2000). Therefore, findings from the Alpha Omega Trial may be extrapolated to the general population.

<u>D3. Intervention</u>

Dosage of n-3 fatty acids
A panel of international experts proposed a minimum daily intake of 200 mg for EPA-DHA and 2 g for ALA (De Deckere 1998). For EPA-DHA, a higher intake of 650 mg/d was recommended during a NIH workshop (Simopoulos 1999). In two groups of the Alpha Omega Trial, dietary EPA-DHA is increased by 400 mg and ALA by 2 g per day. Supplementation with 400 mg EPA-DHA is equivalent to an additional serving (100-150 g) of fatty fish per week (Oomen 2000, Hepburn 1986). In the Zutphen Study of Dutch men aged 64-84 years, the daily intake of n-3 fatty acids was 200 mg for EPA-DHA (<0.1 energy%) and 1.3 g for ALA (0.5 energy%) (Oomen, personal communication). In supplemented subjects of the Alpha Omega Trial, total EPA-DHA intake will be around 400-800 mg/d (0.3 energy%) and total ALA intake around 3.0-3.5 g/d (1.5 energy%). Based on data from observational and experimental studies of n-3 fatty acids (see section B: Background and Significance), these relatively low doses should be sufficient to establish the potential protective effect on coronary heart disease mortality. Subjects are asked to keep their fish intake constant during the trial and to avoid use of fish oil capsules or other n-3 supplements.

Trial margarines
Data from the Third Dutch National Food Survey (Hulshof 1998) in 1997/1998 showed that subjects of 65 y and over consume around 50 g of butter or margarine per day. Part of this amount is used for baking. Daily bread intake in the elderly is around 4 slices in men and 3 slices in women (Hulshof 1998). Use of 6 g of margarine or butter per slice of bread yields an average daily consumption of these spreads of 21 g/d. For the present study, subjects are asked to consume 20 g of trial margarine per day for a period of 3 years. The trial margarines are intended for use on bread, not for baking or food preparation because of spattering and off-flavors

(although the latter is unlikely to occur). Margarines for the trial are produced at regular intervals by Unilever, Vlaardingen, The Netherlands. A frequently consumed margarine available on the Dutch market serves as the basis for trial margarines. Enriched margarines and placebo are of identical taste and appearance to ensure double-blind conduct of the trial. Treatment codes cannot be derived from margarine tubs. After a 4-6 week run-in period on placebo margarine, trial margarines (8 tubs of 250 g) are delivered free of charge to subjects' homes every 12 weeks by cooled courier transport (Valid Express, Amsterdam), under supervision of a specialized logistics company (LOF Logistiek, Egmond aan den Hoef). A food label is printed on margarine tubs with information shown in Table 6. Margarines of active treatment arms contain 400 mg of EPA-DHA (derived from fish oil: Marinol®) and/or 2 g of ALA per 20 g to achieve the required supplementation levels for the trial. The 'neutral' oleic acid is exchanged for n-3 fatty acids. Margarines for all four trial arms are comparable for concentration of linoleic acid. There is at present little scientific evidence for an effect of oleic acid *per se* on coronary mortality.

Table 6. Composition of trial margarines, per 100 g product*

Energy	2990 kJ (730 kcal)
Protein	0 g
Carbohydrates	0 g
Fat	80 g
-Saturated	17 g
-Unsaturated	63 g
Fiber	0 g
Sodium	0 g
Vitamin A	800 μg (100% RDI)
Vitamin D	7.5 μg (150% RDI)
Vitamin E	40 mg (400% RDI)
Depending on type of trial margarine:	
ALA	10 g (exchanged for oleic acid)
EPA-DHA (ratio 5:4)	2 g (exchanged for oleic acid)
Other ingredients: water, mono and diglycerides (E471; emulsifier), potassium sorbate (E202; preservative), citric acid (E330; acidulant), EDTA (E385; stabilizer), flavoring, natural carotenes (E160a; coloring agents)	

*Developed and produced by Unilever, Vlaardingen, The Netherlands, specifically for the Alpha Omega Trial; RDI: Recommended Daily Intake.

Safety issues

Alteration of the EPA-DHA intake in this trial yields an average daily intake of 600 mg/d (200 mg habitually consumed + 400 mg from margarines), which is still lower than, for example, the daily intake of 750 mg in Portuguese fishermen (Torris 2000). In 1997, the US Food and Drug Administration (FDA) approved supplementation with fish oil up to 3.5 g daily for inclusion in the GRAS ('Generally Regarded As Safe') list. Neither the DART-trial (Burr 1989) that increased dietary intake of fatty fish nor the GISSI-trial (GISSI Prevenzione Investigators 1999) that provided higher doses of fish oil found serious side effects. In the GISSI-trial the occurrence of minor side-effects were a reason for discontinuing therapy in 3.8% of subjects in the fish oil group, with gastrointestinal disturbances (4.9%) and nausea (1.4%) as the most frequent complaints. Although the low doses of n-3 fatty acids in trial margarines are unlikely

to cause health problems, serious adverse events are monitored in the Alpha Omega Trial since unanticipated events may occur. A recent meta-analysis suggested a positive association between intake or blood levels of ALA and risk of prostate cancer (Brouwer 2004). Although there is doubt on a causal link (Willett 1997), the occurrence of prostate and other cancers will be carefully monitored during the trial.

D4. Data collection

Invitation of participants and screening
A total of 18 Dutch hospitals are included in this multicenter trial. Eligible patients are contacted by their own cardiologist by means of a letter with which an information brochure is enclosed. Subjects can express their interest in participation by returning a postage-paid reply card either to the Alpha Omega Coordinating Center at Wageningen University or the hospital cardiology department. Subsequently, a telephone screening is performed either by a research nurse or a member of the research team who uses a brief computerized questionnaire on fish consumption, margarine use, use of n-3 supplements, history of myocardial infarction, presence of severe diseases, and a number of other in/exclusion criteria.

Baseline examination
Subjects who meet the inclusion criteria are examined by a research nurse, either at home or at the clinic. Research nurses who visit patients at home are appointed to regions with a radius of approximately 50-km. Before the baseline visit, subjects fill out the Lifestyle and Health and Food Frequency Questionnaire (see Appendix) that they receive by postal mail. The 2-hr baseline examination by the research nurse comprises the following procedures:

- Detailed explanation of the trial and signing informed consent
- Check of personal details (name, address, phone number, e-mail, date of birth) and details of close relative/friend, general practitioner, and cardiologist
- Check of questionnaire data (medical history, use of medication, physical activity, smoking pattern, alcohol use, diet)
- Mini-Mental State Examination (MMSE) for cognitive functioning
- Physical examination (body weight, height, waist circumference, blood pressure)
- Blood sampling (3 tubes)

Physical activity is assessed by the self-administered Physical Activity Scale for the Elderly (Washburn 1993). Dietary intake during the past month is assessed by a comprehensive self-administered food frequency questionnaire (see Appendix). Patterns of current and past smoking (type, amount, frequency, duration, age at start, passive smoking) and alcohol use (type, amount, frequency, week/weekend drinking, binge drinking) are assessed by questionnaires. The Mini-Mental State Examination (MMSE) is performed as a test of cognitive function. Patients with cognitive decline (score ≤ 21) are excluded to avoid difficulties with trial procedures and dietary recall. Body weight (kg) and height (cm) are measured without shoes, with the subject wearing light indoor clothing. The body mass index is computed as height divided by weight squared (kg/m^2). Waist circumference (cm) is measured at the smallest point between the bottom rib and the top of the hipbone using a flexible, non-elastic tape. Blood pressure is measured twice with a 1-minute interval on the left upper arm in sitting position, using an automated device (Omron HEM-711). A larger cuff is used if arm circumference exceeds 32 cm. Heart rate is recorded automatically.

Validation of the Food Frequency Questionnaire

The Food Frequency Questionnaire (see Appendix) has been developed for the older population of the Alpha Omega Trial and is a modified version of an existing Dutch dietary questionnaire that has extensively been validated for total fat intake and different fatty acids (Feunekes 1993). Items have been added for adequate assessment of EPA-DHA intake (fish species, based on fat content) and intake of ALA-rich foods (e.g. nuts, specific oils and green leafy vegetables). Validation for n-3 fatty acids and fish intake is conducted during the Alpha Omega Trial, using the fatty acid composition of plasma cholesterylesters as a biomarker of intake (reflecting the past month). Plasma fatty acids are assessed at baseline and after 1.5 and 3 y of intervention in 800 randomly selected subjects. Women are deliberately oversampled to achieve a gender ratio of 400:400, with equal distribution of subjects over the 4 treatment groups. Relevant questions of the FFQ (e.g. fish intake, margarine use, and use of n-3 supplements) are also incorporated in the yearly telephone interviews.

Data management and quality control

Data are collected by research nurses who are well-experienced with standardized data collection for clinical trials. Before research nurses start their activities, they undergo a one-day training session by the Logistics Manager of the Alpha Omega Trial. Data collection is performed under protocolized conditions, as described in the document 'Standard Operating Procedures of the Alpha Omega Trial' (see Appendix). Time deviations from the protocol within clinical sites is not likely to occur, since most centers recruit patients for the trial during a relatively restricted time period (~1 year). Adherence to SOP is checked continuously by the Logistics Manager who has direct on-line access to all data entered by research nurses in a central database (via www.alphaomegatrial.com). Furthermore, a Data Quality Monitor is fully appointed to the trial who, together with the Logistics Manager, visits clinical sites every month. Research nurses involved in the Alpha Omega Trial attend a meeting with the research team twice per year, during which the SOP is again presented. Whenever inaccuracies in data collection are notified, research nurses are contacted immediately and the SOP is reinforced. In the database of the Alpha Omega Trial, data are linked to research nurses, i.e. it is known by whom a patient had been examined. Nurses' identity codes are registered with all measurements and will be added to multivariate models to adjust for interrater differences, should these occur. Completeness and accuracy of dietary questionnaire data and other data are not only checked by research nurses but also by the Data Quality Monitor (who is a nutritionist by training) at the Alpha Omega Coordinating Center at Wageningen University. Subjects are contacted by telephone within 2 weeks after the baseline examination if self-administered questionnaires do not meet predefined standards, for example if key questions have been left blank.

Data are stored in a single organized SQL database developed by ThePractise.com, Leusden, The Netherlands (www.thepractise.com). Selected parts of the database are accessible by research nurses, research staff, logistics companies, and patients. Data entry at different clinical research sites take place over a secure internet connection to the secure network host of ThePractise.com. Data entry (with double entry checks) occurs under direct supervision of the Trial Assistant and Logistics Manager, and under overall responsibility of the Trial Coordinator at Wageningen University. The internet-accessible database is thus used to facilitate logistics, to check completeness and uniformity of data collection, and to monitor the progress of the Alpha Omega Trial. Randomization tables are stored in a

safe under supervision of a third person at Wageningen University who is otherwise not involved in the trial. Treatment codes remain sealed for research staff and committee members of the Alpha Omega Trial during the course of the trial, except for the DSMB in case of safety matters.

Blood sampling
Venipuncture is performed in all 4000 subjects at baseline. Blood is collected in 3 different PET tubes, namely (1) 10-ml tube with 18 mg (K2 dry) EDTA to prevent polyunsaturated fatty acids from iron-catalyzed oxidation, especially in hemolyzed plasma samples, (2) 4.0-ml tube with 8 mg potassium oxalate and 10 mg sodium fluoride, as antiglycolytic agents, and (3) 9.5-ml serum separator (SST) tube. Plastic, tight-fitting, sealed packages are used to send blood tubes by regular postal mail to the central laboratory at the National Institute for Public Health and the Environment (RIVM), Bilthoven, where blood samples are handled and stored. Standard laboratory determinations include serum total and HDL-cholesterol, serum triglycerides, and plasma glucose. In addition, at baseline and after 1.5 y and 3 y of intervention, the fatty acid composition of plasma cholesteryl esters is examined in 800 randomly selected subjects. Subjects are asked to avoid a heavy meal before venipuncture and the number of hours after breakfast or lunch is recorded. Plasma, serum and buffy coat (DNA) aliquots are stored for at –80°C at the RIVM laboratory. Ideally, there is no more than 24 h delay between blood sampling and handling (freezing). The efficacy of the blood handling protocol and how it influences stability and reliability of blood parameters was tested before the start of the trial. We showed that measured concentrations of fatty acids in plasma cholesteryl esters, specifically the n-3 series, were stable and highly reliable, even after a 24-h delay in processing (details in section C2: Blood sampling procedure; Giltay *et al*, Clin Chem 2003, see Appendix). Laboratory determinations are performed at RIVM, except for plasma fatty acids which are measured at the Division of Human Nutrition, Wageningen University. Standard kits (Roche Diagnostics, Basel, Switzerland) and an autoanalyzer (Hitachi 912, Roche Diagnostics, Basel, Switzerland) are used to determine lipids and glucose. LDL cholesterol is calculated by the Friedewald formula.

Endpoint registration
Events are coded according to the International Classification of Diseases, 10th revision (ICD-10, WHO 1992). Coding is performed by the Endpoint Adjudication Committee (EAC) that consists of three cardiologists and an epidemiologist (chair: Dr Deirdre van der Kuip, MD). A continuous update of the vital status of trial participants is provided by the National Institute for Public Health and the Environment (RIVM), Bilthoven, via a computerized link with the municipal health registries in the Netherlands. A 100% follow-up for vital status is achieved. For coding of endpoints, death certificates, hospital discharge letters and subjects' records are obtained from cardiologists and general practitioners. The primary endpoint of the trial is mortality from CHD during the 3-year trial period. This endpoint category comprises ICD-10 codes I20-I25 and sudden cardiac death. Sudden cardiac death includes ICD-10 code I46 (cardiac arrest) and non-specified death (ICD-10 codes R98-R99) for which a cardiac cause cannot be excluded, occurring within 12 h after the onset of symptoms. Secondary endpoints comprise all-causes mortality and total cardiovascular mortality, which includes fatal cerebrovascular disease (ICD-10 codes I60-I69), during the 3-year trial period.

Follow-up for non-fatal events and cardiovascular health
Participants are asked to record all hospitalizations and potential side effects of n-3 supplementation, including gastrointestinal problems, in a structured patient diary. In addition, participants inform research staff about hospitalizations (as well as change of address, institutionalization, and vacations) by means of postage-paid reply cards. Information about non-fatal myocardial infarctions, coronary interventions, and other health-related events is performed by research staff who contacts all participants by telephone after 1, 2 and 3 years of intervention. A structured protocol is used for collection of follow-up data, making use of a computerized questionnaire in which information from previous patient diaries is incorporated (see Appendix, #9 and #10). During these telephone interviews (duration: approx. 20 minutes), information is obtained on cardiovascular health, diabetes mellitus, gastrointestinal health, malignancies, hospitalizations (for any cause), self-reported body weight, changes in medication, use of dietary supplements, and current fish consumption. Subjects also report current intake of margarine and whether they are satisfied with the margarine.

Baseline measurements and blood sampling are repeated in a randomly selected group of 800 subjects after 1.5 y of intervention to monitor changes in lifestyle (alcohol use, smoking, physical activity, etc.), diet (by means of food frequency questionnaire), medication, anthropometry and cardiovascular health (serum lipids, serum glucose, blood pressure). The total cohort will undergo final examination by research nurses after 3 y of intervention, similar to baseline measurements. Around 3000 subjects will be re-examined after 3 y, given the anticipated number of 600 drop-outs and (at least) 400 deaths during the trial.

Monitoring of compliance
Compliance to intervention in the Alpha Omega Trial is assessed as follows:
1) Registration of unopened tubs of previous margarine deliveries.
2) Check on margarine use and satisfaction with trial margarine by research staff after 1, 2 and 3 years of intervention during a structured telephone interview.
3) Patient diaries, in which participants register codes that are printed on margarines tubs and report any irregularities in margarine use.
4) Objectively, by measurement of fatty acid composition in plasma cholesteryl esters (Zock 1997) in random subgroups of 800 patients at baseline and after 1.5 and 3 y of intervention. Margarine tub count will be validated against plasma n-3 fatty acids to deduce the degree of compliance for all subjects. A high level of compliance is expected on basis of pilot experience, excellent quality and taste of trial margarines and the fact that margarine use on bread is very common in the Netherlands (even more so at old age). Apart from intention-to-treat analysis, a *per-protocol* analysis will be performed in compliant subjects, based on (validated) tub count.

Loss to follow-up
For vital status, a 100% follow-up is achieved by the computerized link with municipal registries. In addition, patients provide contact details of a close relative or friend (not a household member) and of the general practitioner and cardiologist from whom information on the subject’s health status (e.g. in case of hospitalization) or other relevant information can be obtained. Subjects are asked to report to the Alpha Omega Coordinating Center when they are hospitalized, admitted to a nursing home or other institution, or in case of vacation. Because of margarine deliveries by courier every 12 weeks, it is always notified when a subject is away from home for a longer period of time so that appropriate action can be taken. If a subject cannot be

contacted by telephone or e-mail during follow-up, a certified letter is sent. In case of no response, the relative or friend is contacted. If communication remains unsuccessful, the subject is considered lost to follow-up (only for non-fatal events).

D5. Time schedule

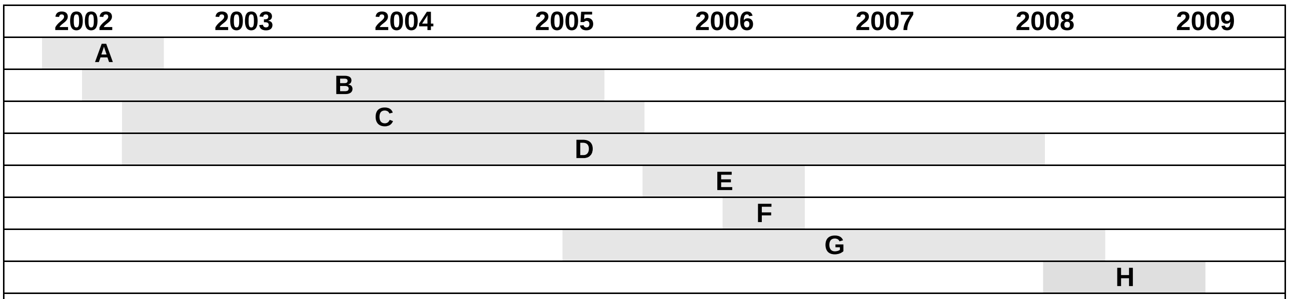

A: Pilot study in first 400 patients
B: Recruitment and screening patients in collaboration with Dutch cardiologists
C: Baseline examinations, 4-6 week run-in period and randomization; measurement of plasma n-3 fatty acids in 800 randomly selected subjects
D: Follow-up (3-y intervention period) with yearly telephone contact, monitoring of vital status and recording of endpoints
E: Re-examination of 800 randomly selected subjects after 1.5 y by research nurses; measurement of plasma n-3 fatty acids in 800 randomly selected subjects
F: Formal interim analysis by Data and Safety Monitoring Board
G: Final examination of total cohort after 3 y by research nurses; measurement of plasma n-3 fatty acids in 800 randomly selected subjects
H: Data-analysis and publication

D6. Statistics

Power considerations
The sample size is based on detecting an effect primarily for EPA-DHA, because for ALA the scientific evidence for a cardioprotective effect is less consistent. Sample size calculation is based on the following data and assumptions:

- Study population: Dutch men and women aged 60-80 y with a history of myocardial infarction
- Primary endpoint: coronary heart disease mortality with an incidence of 4% per year (exclusive of in-hospital mortality) in the placebo group.
- Trial duration: 3 years
- Relative risk of coronary death for EPA-DHA supplementation: 0.75
- Power of 80% and significance level of 5%

The required sample size for demonstrating an effect of EPA-DHA would be 3408 subjects. A larger sample size of 4000 subjects was taken for the Alpha Omega Trial to account for a potential dropout rate of 5% per year. The trial has a power of 67% for detecting an effect of ALA under the assumption of a relative risk of 0.80 for coronary heart disease mortality. The expected number of primary endpoints (coronary deaths) in different treatment groups is given in Table 7.

Table 7. Expected number of coronary deaths in the Alpha Omega Trial

Treatment	*n*	Coronary mortality (%)		No. of coronary deaths
		1 year	3 year	
EPA-DHA-placebo + ALA-placebo	1000	4.0	12.0	120
EPA-DHA + ALA-placebo	1000	3.0	9.0	90
ALA + EPA-DHA-placebo	1000	3.2	9.6	96
EPA-DHA+ALA	1000	2.2	6.6	66
Total	4000	3.1	9.3	**372**

Data-analysis

Double-data entry is performed, except for patient diaries. Continuous data are checked for outliers, which are verified manually against patient records. Database records are created on basis of randomization numbers, such that patients cannot be identified on basis of demographic characteristics. Treatment codes will be merged to the database when all other data have been approved. Discrete variables are analyzed using Chi-square or Fisher's exact test, depending on the distributions, and continuous variables by linear regression. Skewed values are log-transformed to achieve normality. Two-sided P-values <0.05 are considered statistically significant.

Data are analyzed according to the intention-to-treat principle, using SAS statistical software. In principle, the Kaplan Meier procedure is used to assess event-free survival for primary and secondary endpoints associated with n-3 fatty acid supplementation. In case that baseline characteristics are not equally distributed among the treatment groups, Cox survival analysis is used with adjustment for potential confounders. Adjustment will be made for interobserver variability by adding identification codes of research nurses and/or clinical center codes to the multivariate models, if necessary. For each endpoint, two analyses are performed to assess the effect of [1] EPA-DHA supplementation, and [2] ALA supplementation, making optimal use of the 2x2 factorial design (Figure 3). An additional *per-protocol* analysis will be done in compliant patients, on basis of validated margarine tub count. Data will be reported as relative risks (hazard ratios) with 95% confidence intervals and two-sided P-values. For subgroup analysis by gender, power is not sufficient. However, based on the literature (He *et al*, Circulation 2004;109:2705-2711), a similar strength of association is expected in women and men. Because of the smaller sample of women, the confidence interval around the estimate will be wider.

Data-analysis for primary and secondary endpoints of the trial is performed by dr. Eric Boersma, who is an independent biostatistician at the Thorax Center at Erasmus Medical Center Rotterdam and also chair of the Data and Safety Monitoring Board (Biosketch included at p. 25 of this Application). Additional analyses using the database of the Alpha Omega Trial will be performed in the year 2009 by the Trial Coordinator, who is a registered epidemiologist, assisted by the Trial Assistant.

Figure 3. Statistical analysis for 2x2 factorial design of the Alpha Omega Trial

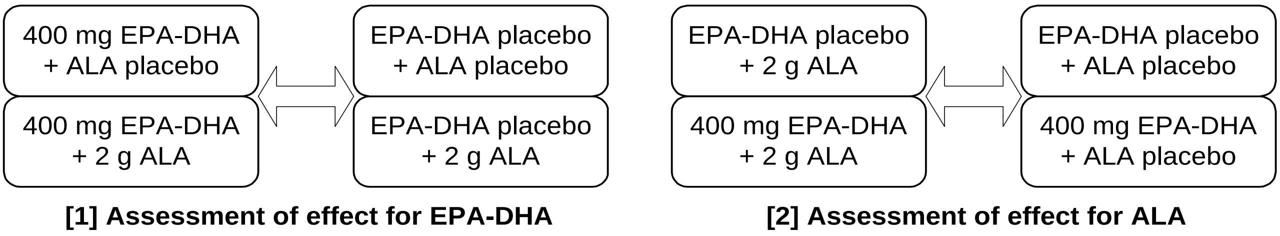

D7. Randomization

Patients are recruited sequentially after which they are randomly assigned to one of four treatments. Simple randomization is applied, using a randomization table (with a randomization ratio of 1:1:1:1). The table was produced on the computer by a random-number generator before the start of the trial, with numbers running from 1001 through 9999. The randomization number also serves as the identification number known to all parties involved in the Alpha Omega Trial, including patients. Treatment codes (A,B,C,D) are assigned by Unilever to four types of trial margarine and are not known to others involved in the trial. A table linking randomization numbers to treatment codes is stored in a safe, which is only accessible by a third person who is not involved in the Alpha Omega Trial.

D8. Trial organization

Executive Committee

Members of the Executive Committee are Dr. Daan Kromhout (Principal Investigator), Dr. Evert Schouten (Co Principal Investigator), and Dr. Marianne Geleijnse (Trial Coordinator). This committee takes care of the day-to-day management, supervises research nurses and other research staff, monitors adherence to the trial protocol in different centers, resolves emerging problems, acquires financial resources, informs the Steering Committee and funding organizations, and takes care of data reporting and scientific publications. The meeting frequency of the Executive Committee is twice a month. Meetings are also attended by the Trial Assistant (Janette de Goede) and Logistics Manager (Annemarie Teitsma-Jansen).

Steering Committee

The Steering Committee is the main policy and decision making board of the trial and has final responsibility for scientific output. Members of the Steering Committee are Dr. Barbara Mulder (cardiologist; Chair), Dr. Wim Saris (nutritionist; Vice Chair), Dr. Jaap Deckers (cardiologist) and Dr. Martijn Katan (biochemist). The members of the Steering Committee are independent, i.e. not otherwise involved in the trial. The committee monitors progress of the trial and ensures that there are no major deviations from the study protocol. Yet, when judged necessary, the committee may decide to modify the protocol for which approval needs to be obtained from the Medical Ethical Committee. The committee ensures that data are properly analyzed, published and presented. The committee may decide on premature termination of the trial if there are severe logistical or financial problems, if subject recruitment goals cannot be met, or if interim analysis indicates significant treatment effects or serious side effects of intervention. The Steering Committee meets twice per year. Meetings are attended by the Executive Committee (no right to vote) and representatives of the Netherlands Heart Foundation (no right to vote) and Unilever (no right to vote).

Endpoint Adjudication Committee (EAC)

The Endpoint Adjudication Committee is responsible for the classification of the primary and secondary endpoints. Members are Dr. Deirdre van der Kuip (general practitioner/epidemiologist, Chair) who has no right to vote, and three independent cardiologists. Members of the EAC code fatal events according to the International Classification of Diseases, 10th revision (ICD-10; WHO 1992). EAC members are blinded to treatment assignment of all patients. Cardiologists will not code events of their own patients, should they participate in the trial. Each case is independently

reviewed and coded by 2 cardiologists. In case of discrepancy, they arrange a consensus meeting. In case that consensus cannot be reached, the entire Endpoint Adjudication Committee discusses the event and the most appropriate ICD-10 code is assigned (if necessary by voting).

E. Human subjects research

- Protection of Human Subjects

The Alpha Omega Trial is conducted in accordance with the Declaration of Helsinki. Prior to any study-specific tests or procedures, the benefits and risks of the study are carefully explained to the subject both orally and in writing. At baseline, an informed consent form is signed. Subjects are allowed to withdraw from the study at any time without specifying a reason. Informed consent also covers retrieval of patients' records at the general practitioner's office and/or hospital and the use of biomaterials (i.e., buffy coat) for DNA analysis. Wageningen University has a contract with an insurance company (St. Paul, www.stpaul.com), should harm or injury to trial participants occur. This dietary intervention study, however, has little impact on normal daily living and health risks are expected to be minimal. During the trial patients remain under control with their own cardiologist and therapy will be continued, including prescribed drugs. An independent general practitioner (Dr. C.J. Fieren, Wageningen, The Netherlands), who is otherwise not involved in the trial, can be contacted by patients should they have problems during the trial that they do not want to discuss with the research team or their own cardiologist. Furthermore, the research team can be contacted by patients, cardiologists and research nurses during 24-hours per day, 7 days per week (mobile telephone help desk).

The trial protocol has been approved by the central authorized Medical Ethics Committee of the Hospital 'Leyenburg', The Hague, at 2 April 2002 (protocol number 01.049, see Appendix), and has been judged by ethical committees of all participating hospitals. In case of protocol modification, the Steering Committee submits a formal amendment to the authorized Medical Ethical Committee prior to implementation. Protocol modifications include changes in study objectives, design, strategy for subject recruitment, treatment regimens, measurements, follow-up procedures and committee tasks and memberships.

The Alpha Omega Trial is compliant with the Personal Data Protection Act by the Dutch government (in Dutch: 'Wet Bescherming Persoonsgegevens'). The Dutch DPA as an independent institution checks that the personal data are used with caution and that citizens' privacy continues to be sufficiently guaranteed, both now and in the future. The Alpha Omega Trial is registered at this institution (no. m1130166). Trial participants are assigned an identity number. Personal details (e.g., name, address, date of birth) are separated from research data and these are stored in different places so that they cannot directly be linked.

- Inclusion of Women

Both men and women in the age group 60-80 y who had a myocardial infarction in the past 10 y are eligible for the Alpha Omega Trial. No selection on basis of gender is made. However, due to the higher prevalence of CHD in men, female participants will probably be less represented in the trial. The pilot study (see section C2, Preliminary Studies) showed that this was indeed the case, because the male:female ratio was 3:1. This will probably also be the case for the remainder of the cohort that is to be enrolled in 2004-2005. The number of postmyocardial infarction patients not

involved in other (pharmacological) trials that meet the inclusion criteria for the Alpha Omega Trial is limited. Therefore, we decided that every eligible male patient could participate in the trial. However, we will deliberately oversample women for the re-examination phase after 1.5 y of intervention, in which 800 randomly selected subjects will participate (gender ratio of 400:400). This enables us to adequately monitor factors related to cardiovascular health in women during the trial. For subgroup analysis of primary and secondary endpoints in women, power is not sufficient. However, based on the literature (He *et al*, Circulation 2004;109:2705-2711), a similar strength of association is expected as in men. Because of the smaller sample of women, the confidence interval around the estimate will be wider.

♦ Inclusion of Minorities

Ethnic minorities form about 9% of the total Dutch population, and about 30% of the population in main cities. The ethnic minority population is dominated by four groups, i.e. Turks, Moroccans and people from the former Dutch colonies of Antilles and Surinam. Since the 1990s the number of refugees various Asian and African countries has grown, and these new immigrants now form nearly 25% of the ethnic minority population. All ethnic groups are eligible for participation in the Alpha Omega Trial. However, patients need to be able to communicate in Dutch and must be willing to consume around 20 gram of margarine per day. Margarine use is not common among Dutch ethnic minority groups. In the pilot study (see section C2, Preliminary Studies) the number of participants from ethnic minorities was very small (1.3%) despite the fact that hospitals in main cities participated. Therefore, we estimate that at maximum 20 women and 60 men in the total cohort (2%) will be from an ethnic minority. In data-analysis, the effect of n-3 fatty acid supplementation cannot be examined for different ethnic groups due to lack of statistical power.

♦ Inclusion of Children

The primary endpoint of the Alpha Omega Trial is coronary heart disease mortality. For reasons of feasibility and efficiency, children cannot be included in the trial.

♦ Data and Safety Monitoring Plan

The Alpha Omega Trial has a Data and Safety Monitoring Board (DSMB) that ensures the safety of the trial and the validity and integrity of the data. Members are Dr. Eric Boersma (biostatistician, Chair), Dr. Wouter Jukema (cardiologist) and Dr. Jan Jaap van Binsbergen (general practitioner). DSMB members are not affiliated with Wageningen University, the National Institute for Public Health and the Environment (RIVM), the Netherlands Heart Foundation or participating hospitals.

As an independent board, the DSMB effectively protects the rights and interests of the study participants. The DSMB is familiar with the trial protocol. Research staff at Wageningen University provides the DSMB with relevant information regarding enrolment, compliance, dropouts, possible adverse reactions, and clinical events at regular intervals. The DSMB monitors the occurrence of serious adverse events, which are defined as ‘hospitalization for any reason that may or may not be causally linked to intervention’. Research nurses provide information on hospitalizations of trial participants to the research staff of the Alpha Omega Trial at Wageningen University if this comes to their attention. Also, research staff is informed at 12-week intervals by the logistics company (LOF, Egmond aan den Hoef, The Netherlands) if patients are not at home for delivery of trial margarines by courier service (Valid Express, Amsterdam). Furthermore, participants are asked to send

postage-paid reply cards to the Alpha Omega secretariat in case they are hospitalized (or not at home for other reasons, e.g. vacation and admission to nursing home). Participants also record hospitalizations and health problems in a patient diary, which is checked once a year by telephone.

The DSMB provides summary reports for feedback at regular and defined intervals to the Steering Committee and is allowed to recommend modifications to the protocol. A formal interim analysis by the DSMB to examine treatment effects on primary and secondary endpoints is scheduled in fall 2006. The DSMB informs the Steering and Executive Committees about the outcome of interim analysis within 2 weeks. A formal decision for premature termination of the trial is made at $p<0.001$ (ALA-placebo and/or EPA-DHA-placebo differences) during interim analysis. For data-analysis, the chair of the DSMB receives the entire database, including treatment codes (A, B, C, and D; triple blinded procedure). The Chair acknowledges receipt and summarizes relevant data in a table, which is then forwarded to other DSMB members. All members judge the data, and a meeting will be arranged to determine what type of action(s) will be advised to the Steering Committee. The DSMB is the only Trial Committee that can have access to the unblinded data, upon request. If significant differences in endpoints or serious adverse events exist among the treatment arms (either in positive or negative direction), the treatment codes may be broken. Decisions are based on consensus and, when necessary, on voting. The meeting frequency of the DSMB is once per year, or more often in case or urgent safety matters. The DSMB will perform the final data analysis on primary and secondary endpoints in 2008, for which treatment codes need to be broken.

F. Vertebrate Animals

Not applicable

G. Literature cited

Albert CM, Hennekens CH, O'Donnell CJ, et al. Fish consumption and risk of sudden cardiac death. JAMA 1998;279:23-8.

Albert CM, Campos H, Stampfer MJ, et al. Blood levels of long-chain n-3 fatty acids and the risk of sudden death. N Engl J Med 2002;346:1113-8.

Bang HO, Dyerberg J, Sinclair HM. The composition of the Eskimo food in north western Greenland. Am J Clin Nutr 1980;33:2657-61.

Brouwer IA, Katan MB, Zock PL. Dietary α-linolenic acid is associated with reduced risk of fatal coronary heart disease, but increased prostate cancer risk. A meta-analysis. J Nutr 2004;134:919-22.

Burr ML, Fehily AM, Gilbert JF, et al. Effects of changes in fat, fish, and fibre intakes on death and myocardial infarction: Diet and reinfarction trial (DART). Lancet 1989;2:757:61.

Burr ML, Ashfield-Watt PA, Dunstan FD, et al. Lack of benefit of dietary advice to men with angina: results of a controlled trial. Eur J Clin Nutr 2003;57:193-200.

De Deckere EAM, Korver O, Verschuren PM, Katan MB. Health aspects of fish and N-3 polyunsaturated fatty acids from plant and animal origin. Eur J Clin Nutr 1998;52:749-53.

De Lorgeril M, Renaud S, Mamelle N, et al. Mediterranean alpha-linolenic acid-rich diet in secondary prevention of coronary heart disease. Lancet 1994;343:1254-9.

Erkkilä AT, Lehto S, Pyörälä K, Uusitupa MI. n-3 Fatty acids and 5-y risks of death and cardiovascular disease events in patients with coronary artery disease. Am J Clin Nutr 2003;78:65-71.

Feunekes GI, Van Staveren WA, De Vries JH, et al. Relative and biomarker-based validity of a food-frequency questionnaire estimating intake of fats and cholesterol. Am J Clin Nutr 1993;58:489-96.

Gann PH, Hennekens CH, Sacks FM, et al. Prospective study of plasma fatty acids and risk of prostate cancer. J Natl Cancer Inst 1994;86:281-6.

Giltay EJ, Geleijnse JM, Schouten EG, et al. High stability of markers of cardiovascular risk in blood samples. Clin Chem 2003;49:652-5.

Giovannucci E, Rimm EB, Colditz GA, et al. A prospective study of dietary fat and risk of prostate cancer. J Natl Cancer Inst 1993;85:1571-9.

GISSI-Prevenzione Investigators (Gruppo Italiano per lo Studio della Sopravvivenza nell'Infarto miocardico). Dietary supplementation with n-3 polyunsaturated fatty acids and vitamin E after myocardial infarction: results of the GISSI-Prevenzione trial. Lancet 1999;354:447-55.

Grundy SM. N-3 fatty acids: priority for post-myocardial infarction clinical trials. Circulation 2003;107:1852-7.

Guallar E, Aro A, Jimenez FJ, et al. Omega-3 fatty acids in adipose tissue and risk of myocardial infarction: the EURAMIC study. Arterioscler Thromb Vasc Biol 1999;19:1111-8.

Guallar E, Inmaculada Sanz-Gallardo M, van ‘t Veer P, et al. Mercury, Fish Oils, and the Risk of Myocardial Infarction. N Engl J Med 2002;347:1747-54.

He K, Song Y, Daviglus ML, Liu K, Van Horn L, Dyer AR, Greenland P. Accumulated evidence on fish consumption and coronary heart disease mortality. A meta-analysis of cohort studies. Circulation 2004a;109:2705-11.

He K, Song Y, Daviglus ML, Liu K, Van Horn L, Dyer AR, Goldbourt U, Greenland P. Fish consumption and incidence of stroke. A meta-analysis of cohort studies. Stroke 2004b;35:1538-42.

Hepburn FN, Exler J, Weihrauch JL. Provisional tables on the content of omega-3 fatty acids and other fat components of selected foods. J Am Diet Assoc 1986;86:788-93.

Hodgson TA, Cohen AJ. Medical care expenditures for selected circulatory diseases: opportunities for reducing national health expenditures. Med Care 1999;37:994-1012.

Hulshof KFAM, Kistemaker C, Bouman M. Third Dutch National Food Survey 1997-1998. TNO Nutrition, report V98.805, Zeist, The Netherlands, 1998.

Kang JX, Leaf A. Antiarrhythmic effects of polyunsaturated fatty acids. Recent studies. Circulation 1996;94:1774-80.

Keli SO, Feskens EJ, Kromhout D. Fish consumption and risk of stroke. The Zutphen Study. Stroke 1994;25:328-32.

Kris-Etherton PM, Shaffer Taylor D, Yu-Poth S, et al. Polyunsaturated fatty acids in the food chain in the United States. Am J Clin Nutr 2000;71(Suppl):179S-88S.

Kris-Etherton P, Daniels SR, Eckel RH, et al. Summary of the Scientific Conference on Dietary Fatty Acids and Cardiovascular Health: Conference Summary From the Nutrition Committee of the American Heart Association. Circulation. 2001;103:1034-9.

Kris-Etherton PM, Harris WS, Appel LJ. American Heart Association. Nutrition Committee. Fish consumption, fish oil, omega-3 fatty acids, and cardiovascular disease. Circulation 2002;106:2747-57.

Kromhout D, Bosschieter EB, De Lezenne Coulander C. The inverse relation between fish consumption and 20-year mortality from coronary heart disease. N Engl J Med 1985;312:1205-9.

Kromhout D, Feskens EJ, Bowles CH. The protective effect of a small amount of fish on coronary heart disease mortality in an elderly population. Int J Epidemiol 1995;24:340-5.

Kromhout D, Katan MB, Havekes L, et al. The effects of 26 years of habitual fish consumption on serum lipid and lipoprotein levels (The Zutphen Study). Nutr Metab Cardiov Dis 1996;6:65-73.

Kromhout D. Fish consumption and sudden cardiac death [editorial]. JAMA 1998;1:65-6.

Kromhout D, Menotti A, Kesteloot H, Sans S. Prevention of coronary heart disease by diet and lifestyle: evidence from prospective cross-cultural, cohort, and intervention studies. Circulation 2002;105:893-8.

Lemaitre RN, King IB, Mozaffarian D, et al. N-3 polyunsaturated fatty acids, fatal ischemic heart disease, and nonfatal myocardial infarction in older adults: the Cardiovascular Health Study. Am J Clin Nutr 2003;77:319-25.

Mozaffarian D, Lemaitre RN, Kuller LH, et al; Cardiovascular Health Study. Cardiac benefits of fish consumption may depend on the type of fish meal consumed: the Cardiovascular Health Study. Circulation 2003;107:1372-7.

Murray CJ, Lopez AD. Alternative projections of mortality and disability by cause 1990-2020: Global Burden of Disease Study. Lancet 1997;349:1498-1504.

Netherlands Heart Foundation. Hart- en vaatziekten in Nederland 1995 - Cijfers over ziekte en sterfte (Cardiovascular Diseases in the Netherlands 1995) [Dutch]. The Hague, 1995.

Oomen CM, Feskens EJM, Räsänen L, et al. Fish consumption and coronary heart disease mortality in Finland, Italy, and The Netherlands. Am J Epidemiol 2000;151:999-1006.

Oomen CM, Ocke MC, Feskens EJ, et al. Association between trans fatty acid intake and 10-year risk of coronary heart disease in the Zutphen Elderly Study: a prospective population-based study. Lancet 2001;357:746-51.

Oomen CM, Ocké MC, Feskens EJM, et al. Alpha-linoleic acid intake is not beneficially associated with 10-year risk of coronary heart disease. Am J Clin Nutr 2001;74:457-463.

Raitt M, Connor W, Morris C, et al. Antiarrhythmic effects of n-3 polyunsaturated fatty acids in survivors of ventricular tachyarrhythmias. Circulation 2003;108:2723.

Simon JA, Fong J, Bernert JT Jr, Browner WS. Serum fatty acids and the risk of stroke. Stroke 1995;26:778-82.

Simopoulos AP, Leaf A, Salem N. Essentiality of and Recommended Dietary Intakes for Omega-6 and Omega-3 Fatty Acids. Ann Nutr Metab 1999;43:127-30.

Sinclair AJ, Attar-Bashi NM, Li D. What is the role of alpha-linolenic acid for mammals? Lipids 2002;37:1113-23.

Siscovick DS, Raghunathan TE, King I, et al. Dietary intake and cell membrane levels of long-chain n-3 polyunsaturated fatty acids and the risk of primary cardiac arrest. JAMA 1995;274:1363-7.

Van Houwelingen AC, Hornstra G, Kromhout D, de Lezenne Coulander C. Habitual fish consumption, fatty acids of serum phospholipids and platelet function. Atherosclerosis 1989;75:157-65

Verschuren WM, Jacobs DR, Bloemberg BP, et al. Serum total cholesterol and long-term coronary heart disease mortality in different cultures. Twenty-five-year follow-up of the seven countries study. JAMA 1995;274:131-6.

Voskuil DW, Feskens EJM, Katan MB, Kromhout D. Intake and sources of a-linolenic acid in Dutch elderly men. Eur J Clin Nutr 1996;50:784-7.

Washburn RA, Smith KW, Jette AM, Janney CA. The Physical Activity Scale for the Elderly (PASE): development and evaluation. J Clin Epidemiol. 1993;46:153-62.

WHO. International Statistical Classification of Diseases and Related Health Problems, 10th revision. Vol. 1. Geneva, 1992.

Willett WC. Specific fatty acids and risks of breast and prostate cancer: dietary intake. Am J Clin Nutr 1997;66(6 Suppl):S1557-63.

Zock PL, Mensink RP, Harryvan J, et al. Fatty acids in serum cholesteryl esters as quantitative biomarkers of dietary intake in humans. Am J Epidemiol 1997;145:1114-22.

H. Consortium/contractual arrangements

None.

I. Letters of Support

Attached hereafter.

J. Product development plan

Not applicable.
